# Thermal Conductivity and Mechanical Properties of Polymer Composites with Hexagonal Boron Nitride—A Comparison of Three Processing Methods: Injection Moulding, Powder Bed Fusion and Casting

**DOI:** 10.3390/polym15061552

**Published:** 2023-03-21

**Authors:** Nu Bich Duyen Do, Kristin Imenes, Knut E. Aasmundtveit, Hoang-Vu Nguyen, Erik Andreassen

**Affiliations:** 1 Department of Microsystems, University of South-Eastern Norway, 3184 Borre, Norway; 2 SINTEF Industry, 0373 Oslo, Norway

**Keywords:** hexagonal boron nitride, thermoplastic polyurethane, injection moulding, powder bed fusion, casting, thermal conductivity

## Abstract

Materials providing heat dissipation and electrical insulation are required for many electronic and medical devices. Polymer composites with hexagonal boron nitride (hBN) may fulfil such requirements. The focus of this study is to compare composites with hBN fabricated by injection moulding (IM), powder bed fusion (PBF) and casting. The specimens were characterised by measuring thermal conductivity, tensile properties, hardness and hBN particle orientation. A thermoplastic polyurethane (TPU) was selected as the matrix for IM and PBF, and an epoxy was the matrix for casting. The maximum filler weight fractions were 65%, 55% and 40% for IM, casting and PBF, respectively. The highest thermal conductivity (2.1 W/m∙K) was measured for an IM specimen with 65 wt% hBN. However, cast specimens had the highest thermal conductivity for a given hBN fraction. The orientation of hBN platelets in the specimens was characterised by X-ray diffraction and compared with numerical simulations. The measured thermal conductivities were discussed by comparing them with four models from the literature (the effective medium approximation model, the Ordóñez-Miranda model, the Sun model, and the Lewis-Nielsen model). These models predicted quite different thermal conductivities vs. filler fraction. Adding hBN increased the hardness and tensile modulus, and the tensile strength at high hBN fractions. The strength had a minimum as the function of filler fraction, while the strain at break decreased. These trends can be explained by two mechanisms which occur when adding hBN: reinforcement and embrittlement.

## 1. Introduction

The miniaturisation and increased processing capacity of electronics components often result in high thermal loading [1,2]. Thermal management therefore plays an important role for the performance and reliability of electronic devices. In addition to efficient heat dissipation, electrical insulation is required for many applications, such as mobile devices and medical devices [1,3]. Hence, thermally conducting but electrically insulating polymer-based composite materials are often used in electronic packaging [1,4].

Polymer-based materials have low density and low cost, and allows for mass production with well-established techniques, such as injection moulding and transfer moulding [1,5]. The polymer matrix in a composite can be a thermoset (e.g., epoxy) or a thermoplastic, including elastomers of either type. Most polymers have low thermal conductivity, typically in the range 0.1–0.5 W/m∙K [5]. Incorporating thermally conductive but electrically insulating inorganic fillers into the polymer matrix is an effective solution for improving the thermal conductivity while maintaining its electrical insulation properties [5,6]. Common fillers are crystalline ceramic materials, either metal oxides (e.g., alumina (Al_2_O_3_), quartz (crystalline SiO_2_)), or non-oxides (e.g., aluminium nitride (AlN), boron nitride (BN), silicon nitride (Si_3_N_4_), silicon carbide (SiC)) [5]. Hexagonal boron nitride (hBN) has received attention due to its high intrinsic thermal conductivity and good electrical insulating properties. The filler hBN has been used for enhancing the thermal conductivity of polymer-based composites, see examples in Table 1.

Hexagonal BN consists of B and N atoms arranged in a honeycomb configuration with a layer structure similar to graphite. Within the layers, there are strong covalent bonds between B and N atoms, while the bonds between layers are weak van der Waals forces [15,16]. The crystal structure of hBN results in platelet-shaped particles. The platelets have high in-plane thermal conductivity of about 300–600 W/m∙K, whereas the through-plane thermal conductivity is in the range 2–30 W/m∙K [4,9,15,16]. Furthermore, hBN is electrically insulating and has a wide band gap (about 5.97 eV). It also has high thermal stability and good mechanical properties [15,16,17]. Due to the strong B–N bonds, hBN is chemically stable, e.g., towards oxidation. However, this makes the functionalisation of hBN challenging [16]. hBN also has good biocompatibility [16,18,19], which is essential in medical applications.

Due to the hBN particles’ shape and anisotropic thermal conductivity, the orientation of the hBN platelets in the polymer matrix affects the thermal conductivity of the composite [5,15]. Particles can be oriented via processing or by using electric or magnetic fields [5,7,15,20,21]. Different processing methods for polymer-based composites have different impacts with regard to distributing and orienting the platelets, as well as dispersing agglomerates and stacks of platelets into single platelets. Hence, in general, the properties of polymer composites are affected by the choice of processing method [22].

Approaches for preparing thermally conductive polymer composites include melt compounding (e.g., followed by injection moulding) and mixing with a resin followed by casting and polymerisation (curing) [1,5,15]. Injection moulding (IM) is one of the most common manufacturing processes for the mass production of polymer or polymer composite parts [1,23]. The hBN platelets are reported to be preferentially oriented with the platelet normal in the through-plane (thickness) direction of injection moulded parts, owing to the shear stress in the IM process [5,15]. In a casting process, composites are produced by mixing fillers into a resin such as epoxy, followed by pouring or injecting the mixture into a mould for curing. The casting of hBN/polymer composites can result in almost randomly oriented platelets [5,8,15].

Powder bed fusion (PBF) is one of the most common 3D printing (additive manufacturing) processes for polymer materials. The feedstock is powder, and thermal energy (e.g., from a laser) selectively fuses regions of a powder bed, layer-by-layer, to fabricate 3D objects [24]. Compared to moulding processes, the principal advantages of PBF and other 3D printing techniques include the fast prototyping and production of personalised parts with complex geometry. Few polymer types are commercially available for PBF. Among the most common are polyamide 12 (PA12) and thermoplastic polyurethane (TPU). Polyamide-based composites are also used in PBF, with glass-based fillers (e.g., glass fibres, glass beads) or carbon-based fillers (e.g., carbon black, carbon fibres, carbon nanotubes, graphite) [24,25,26]. There is a growing interest in developing new materials and composites for PBF, as well as the optimisation of PBF processing parameters.

There are several articles about PBF of polymer composites with thermally and electrically conductive fillers (e.g., carbon-based fillers such as carbon fibres [27], graphite [28], CNT [29] and graphene [30], or metal fillers such as Cu [31] and Ag [32]). On the contrary, the literature on PBF with thermally conductive and electrically insulating polymer-based composites is sparse, and most studies use PA12 as the polymer matrix. Yang et al. [11] studied PBF of hBN/PA12 composites, for which co-powders were prepared by combining solid-state shear milling and cryogenic pulverisation [11]. Yuan et al. [12] investigated the effect of incorporating Al_2_O_3_ and hBN on thermal and mechanical properties of PA12 composites processed by PBF. Hon et al. [33] examined the effects of processing parameters on the mechanical properties of the PBF composites containing PA12 and SiC. Zhang et al. [13] combined AlN and hBN for enhancing the thermal conductivity of TPU composites processed by PBF.

This research was motivated by our previous study on the encapsulation of interventional medical devices [34]. Encapsulation materials used in the human body must meet several requirements, such as good heat transfer, electrical insulation, mechanical integrity, biocompatibility, and in some cases, a ‘soft touch’ [3,34]. To achieve a significant increase in thermal conductivity in polymer-based composites, a high loading of inorganic fillers (e.g., hBN) is generally required. However, this normally results in increased hardness [5,15] and reduced ductility. To compensate for this, a soft polymer can be used as the matrix material.

Thermoplastic polyurethane elastomers (TPU) are soft materials based on a block copolymer containing hard and soft segments, and where the former act as physical crosslinks for the soft segments. TPU offers high elasticity over a broad temperature range and high wear resistance [24,26,35]. It is commonly used for PBF and IM, and its suitability for biomedical applications has recently been highlighted [25]. Therefore, the TPU “Ultrasint TPU 88A” (in the form of powder for PBF) was selected as the matrix for composites processed by PBF and IM in this study.

This article focuses on comparing the thermal conductivities of hBN/polymer composites processed by three methods, IM and PBF (with TPU as matrix) and casting (with epoxy as matrix). To the best of our knowledge, this is the first study that has processed TPU with hBN using PBF, with only a mixture of the two powders. The measured thermal conductivities are interpreted using platelet orientation measurements (X-ray diffraction), and by comparing with models by Nan et al. [36], Ordóñez-Miranda et al. [37], Sun et al. [38], and Lewis-Nielsen et al. [39]. These models were included as tools to help in understanding the experimental data, especially how the thermal conductivities of the composites are affected by the fraction and orientation of the hBN platelets and the nature of the interphase between the platelets and the polymer matrix. The reason as to why we have included four models and not simply one is that they are based on different assumptions and provide different predictions. Hence, a comparison of the models has a value in itself. Analysing our experimental data using these models provides a deeper understanding of the models and their limitations.

Thermally conductive composites for demanding applications (e.g., the encapsulation of a medical device in our case) also need good mechanical properties. Hence, the study also includes an assessment of the mechanical properties (hardness, tensile modulus, tensile strength, and strain at break) of the composites. Typically, the hBN fraction in the composite must be a compromise, because increasing the fraction increases the thermal conductivity, but also reduces the ductility of the material, and has a negative effect on the processability via higher viscosity.

## 2. Materials and Methods

### 2.1. Materials

The materials used in this study are summarised in Table 2. The polymer matrix was thermoplastic polyurethane (TPU) for injection moulding and powder bed fusion (3D printing) and epoxy for casting.

### 2.2. Specimen Preparation

**Figure 1:**
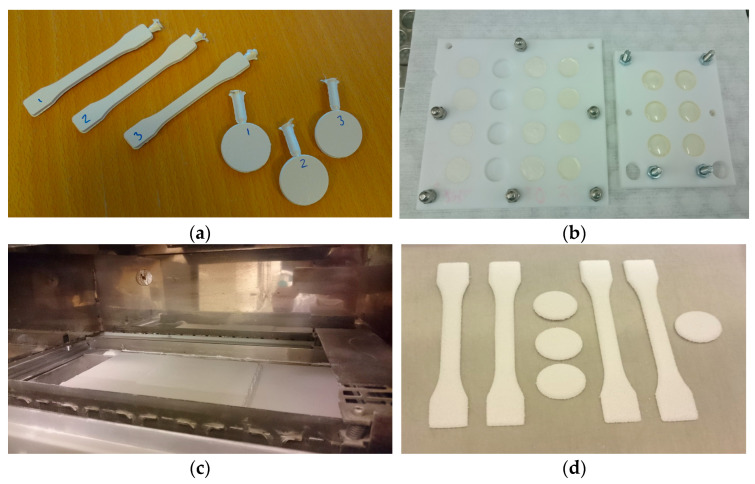
(**a**) Injection moulded specimens (IM_65BN3); (**b**) cast specimens in Teflon moulds; (**c**) PBF 3D printer; and (**d**) PBF specimens.

Injection moulded specimens were prepared using a 15 cm^3^ micro batch compounder (DSM Midi 2000) followed by injection moulding with a table-top machine (DSM). TPU and hBN powders were mixed in the compounder for 3 min in nitrogen atmosphere, and then injection moulded with melt temperature 220 °C and mould temperature 40 °C. Moulded specimens for thermal conductivity measurement were 2 mm thick discs with diameters of 25 mm, while specimens for tensile testing were 2 mm thick and 75 mm long (ISO 527-2, type 1BA), see Figure 1. The maximum practical filler content for this processing route was about 65 wt%. Details about the injection moulded specimens are presented in Table S2 in the Supplementary Materials.

Cast epoxy-hBN specimens were prepared by vacuum mixing, followed by casting into a Teflon mould (Figure 1) and then curing at 150 °C for 18 h. The epoxy system contains 35 wt% bisphenol-F epoxy resin, 35 wt% diluent and 30 wt% curing agent. This system has a low viscosity, which is suitable for high filler concentrations. At the highest filler content (55 wt%), there was no formation of voids or air bubbles during mixing and casting. To have clean and planar surfaces, the cast specimens (2 mm thick discs with diameters of 13 mm) were grinded and polished on both sides. Details about cast specimens are presented in Table S2 in the Supplementary Materials.

For powder bed fusion (PBF), a tabletop PBF 3D printer (Sharebot SnowWhite) with a CO_2_ laser was used to fabricate specimens of a (TPU and hBN) powder mixture. The density and mechanical properties of PBF parts are related to the laser energy density per volume, *E_V_*, which is defined as:

(1) $$E_{V}=\frac{P}{\mathrm{vdL}}$$

where *P* (W) is the laser power; *v* (m/s) is the laser scanning speed; *d* (m) is the hatching distance (the distance between two adjacent laser scan lines); and *L* (m) is the layer thickness. *E_V_* (J/m^3^) represents the amount of energy supplied to a volume element of the powder bed. In order to identify appropriate processing parameters for the hBN/TPU composites, the starting point was the parameters suggested for the pure TPU by the material supplier. However, a higher energy was needed with the printer used, and similarly for the pure TPU. Regarding the chamber temperature, the same settings were used for TPU with and without hBN, because the hBN did not have a large effect on the melting and crystallisation temperatures, see the DSC results in Section S8 in the Supplementary Materials. Details about the printing parameters are provided in Section S2 of the Supplementary Materials.

Most of the PBF specimens in this study were built “flat” (Figure 1), i.e., with the largest surfaces parallel to the plane of the powder bed (the XY plane; the powder recoater moves along the X-axis, and the powder bed moves along the Z-axis). Some specimens were also built “standing”, i.e., with the surface normal in the XY plane. Before printing, the powder was dried in a vacuum oven at 50 °C for 3 h to remove moisture. The maximum filler content used in the PBF process was 40 wt%. At higher hBN loading, the printed parts did not have sufficient cohesion to be used for tensile testing or thermal conductivity measurements. Details regarding the specimens are presented in Tables S2 and S3 in the Supplementary Materials.

### 2.3. Characterisation of hBN/Polymer Composites

#### 2.3.1. Thermal Conductivity Measurement

The thermal conductivity of 2 mm thick specimens was indirectly determined by the non-contact, transient laser flash analysis (LFA) method [41], using the equation:

(2) $$k=\alpha \cdot C_{p}\cdot \rho$$

where *k* is the thermal conductivity (W/m·K); *α* is the thermal diffusivity (mm^2^/s); *C_p_* is the specific heat capacity at constant pressure (J/g·K); and *ρ* is the mass density (g/cm^3^).

The thermal diffusivity was determined at 30, 50, 80 and 110 °C with an LFA instrument (LFA 457 MicroFlash, Netzsch GmbH, Selb, Germany). All specimens, including a reference (Pyroceram 9606), were spray coated with a thin layer of graphite (Graphit 33, Kontakt Chemie, Zele, Belgium) on both sides to reduce reflection, maximise heat absorption and ensure similar surface properties. The thermal diffusivity was determined by the analysis software of the LFA instrument (Netzsch Proteus Software 6.3) using the radiation model without pulse correction.

A Pyroceram reference (diameter 13 mm, thickness 2.5 mm) with known *C_p_* [42] was measured simultaneously, under the same conditions as the composite specimens. Each measurement of a composite specimen was compared to the reference using the analysis software to estimate the unknown *C_p_* value of the composite. Average *α* and average *C_p_* at each temperature were then used for calculating *k* at that temperature.

The specimen dimensions were measured at room temperature using a digital calliper. The thickness and diameter were averaged over five measurements and used for calculating the volume. Each specimen was weighed using a balance with 0.1 mg resolution to calculate the mass density *ρ*. The density of some specimens was also measured gravimetrically (XS204 with density kit, Mettler Toledo, Greifensee, Switzerland). The difference between this density and the density calculated from mass and volume was negligible.

LFA measurements were repeated three times for each specimen at each temperature. Furthermore, for injection moulded specimens, two specimens were measured for each filler concentration, while for PBF and casting, only one specimen was measured for each filler concentration. The variance was dominated by the measurement error (which was about 5–10% at 30 °C, and lower at higher temperatures), not the variation from specimen to specimen.

#### 2.3.2. Tensile Testing

Tensile testing of injection moulded specimens was performed at room temperature, following the standard ISO 527, with a universal test machine (Zwick Z250, Ulm, Germany), using a 2.5 kN load cell. The crosshead speed was 0.5 mm/min up to a strain of 0.25%, and then changed to 25 mm/min (as suggested in ISO 527-1:2012). The tests were run with a slow crosshead speed initially for the accurate determination of the tensile modulus. These speeds were chosen in order to obtain nominal strain rates similar to those typically used in the tensile testing of plastics, with the most common (larger) test specimen 1A of ISO 527-2. PBF specimens were tested with a smaller machine (Lloyd Instruments LR50K, Bognor Regis, UK) without an extensometer. Average values for three to five tests are reported, and standard deviations are included in figures. The stress reported in this paper is the engineering stress, i.e., force divided by the initial cross section. An example of the repeatability of the tensile test is shown in Figure S3 in the Supplementary Materials.

#### 2.3.3. Hardness Measurement

The hardness (Shore A and Shore D) of the specimens were measured with a durometer (Bareiss digi test II, Oberdischingen, Germany) at room temperature, following the standard ASTM D2240. Three specimens were stacked to have the thickness required for the measurements. At least three measurements were performed for each filler content. Hardness measurements were not applicable to the PBF specimens due to high surface roughness.

#### 2.3.4. X-ray Diffraction (XRD)

The orientation of the hBN particles was characterised by X-ray diffraction (XRD), using a Thermo Fisher Equinox 1000 diffractometer (monochromatic Cu Kα radiation; wavelength 1.5418 Å). The operating condition was 40 kV voltage and 30 mA current.

The angle between the specimen surface and the incoming X-ray beam was kept constant at 13.3° (the tilt angle: chosen as half the *2θ* value of one of the peaks used.) Hence, the length of the X-ray beam path from the front surface to the back surface of a 2 mm thick specimen is:

(2 mm)/sin(13.3°) = 8.7 mm

The linear X-ray absorption coefficients of the TPU/hBN composites are typically in the range 8–10 cm^−1^ (increasing with increasing hBN loading). Note that these are only estimates, as we do not know the element composition of the TPU. If the incoming beam intensity is set to unity, and we consider the peak with *2θ* equal to two times the tilt angle, the detected diffracted intensity originating from the centre of a 2 mm thick specimen is simply:

exp(−9∙0.87) = 4∙10^−4^

For comparison, the diffracted intensity from depths of 0.1 mm and 0.3 mm would be about 0.50 and 0.10, respectively, or about 1150 and 240 higher than that from the centre. Furthermore, depending on the beam diameter and the detector slits, the diffraction from deep layers may not be detected at all.

In the diffractograms, the peaks at about 26.6° and 41.5° are the (002) and (100) reflections of hBN. If we assume perfect hBN platelets, the ratio of these two peaks can be used to determine the orientation of the platelets, using Equations (3) and (4) [9,43]:

(3) $$\langle \cos^{2}\theta \rangle =\frac{1}{1+2K}$$

(4) $$K=n\frac{I_{\left(100\right)}}{I_{\left(002\right)}}$$

In these equations *I*_(100)_ and *I*_(002)_ are the integrated intensities of the (100) and (002) peaks, respectively [44], and *n* is a normalisation coefficient, determined to be 6.25 by Tanimoto et al. [9]. Note that we have not corrected *n* for the constant tilt angle of our experiment. In this paper, the degree of orientation of hBN platelets in the polymer matrix is described by $\langle \cos^{2}\theta \rangle$, where $\langle \cos^{2}\theta \rangle$ denotes the average of all platelets, and *θ* is the angle between a platelet surface normal and the specimen surface normal (i.e., the vector along the thickness direction of the specimen). Hence, perfect out-of-plane, in-plane and random orientation of hBN platelets in the specimen corresponds to $\langle \cos^{2}\theta \rangle$ = 0, 1 and 1/3, respectively.

## 3. Results

### 3.1. Thermal Conductivity of hBN/TPU and hBN/Epoxy Composites

**Figure 2:**
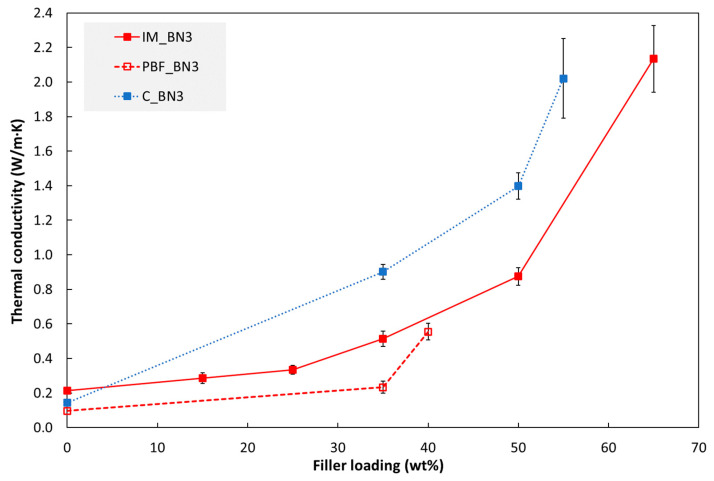
Thermal conductivity (at 30 °C) of composites fabricated by injection moulding (“IM”), powder bed fusion (“PBF”) and casting (“C”) as a function of hBN (BN3) loading. The PBF specimen with 40 wt% hBN was processed with a higher laser energy density than the other PBF specimens in this figure, see Figure 3 and Table S3 in the Supplementary Materials.

Figure 2 shows the thermal conductivity of hBN/polymer composites fabricated by injection moulding (IM), casting (C) and powder bed fusion (PBF) as a function of hBN content. The highest conductivity (2.14 W/m∙K) was measured for an injection moulded specimen with the highest hBN fraction in this study (65 wt%). This conductivity was 9.7 times higher than that of the pure TPU (injection moulded reference). For the cast composites, a conductivity, which was 14 times higher than that of the pure epoxy, was obtained with 55 wt% hBN. For a given hBN content, the cast composites had the highest conductivity and the highest increase relative to the unfilled material. Among the PBF composites, the specimen with 40 wt% hBN, processed with the highest laser energy density (see Table S3 in the Supplementary Materials for more details), had the highest thermal conductivity, which was 5.8 times higher than that of the pure TPU (fabricated by PBF).

**Figure 3:**
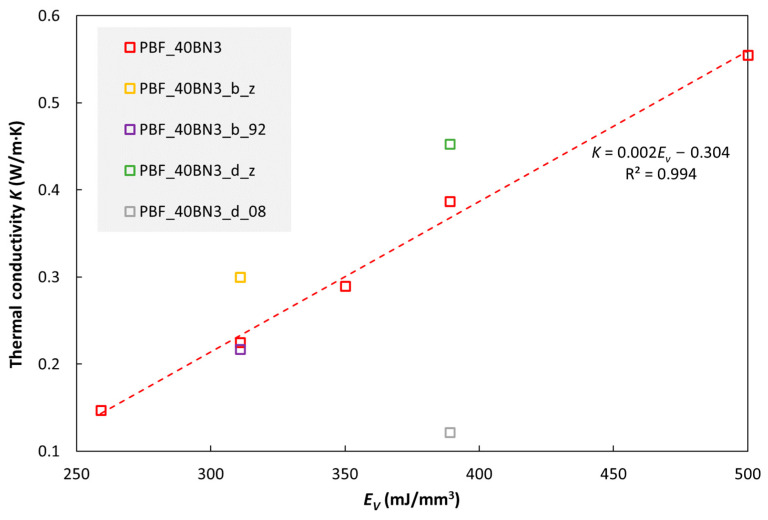
Thermal conductivity (at 30 ºC) of TPU with 40 wt% hBN (BN3) as a function of E_V_ (laser energy density, see Equation (1)). For details about the specimens, see the main text and Table S3 in the Supplementary Materials.

Figure 3 shows the thermal conductivity of PBF composites with 40 wt% hBN as a function of PBF laser energy density (*E_V_*, see Equation (1)). For the main data series in this figure (PBF_40BN3; red open squares), *E_V_* was increased by increasing the laser power or decreasing the scanning speed. There is a clear increase in conductivity with increasing laser energy density for this data series. In addition to the main data series, four single points are also included in the figure, representing other process variations: increasing the nominal *E_V_* by reducing the layer thickness from 0.1 to 0.08 mm resulted in a reduction in conductivity (specimen PBF_40BN3_d_08). Specimens that were built along the z direction of the 3D printer (specimen codes ending in “_z”) had higher conductivity than those that were printed “flat” in the XY plane (all the other data points in the figure). Increasing the chamber temperature from 88 °C to 92 °C had no significant effect (PBF_40BN3_b_92).

**Figure 4:**
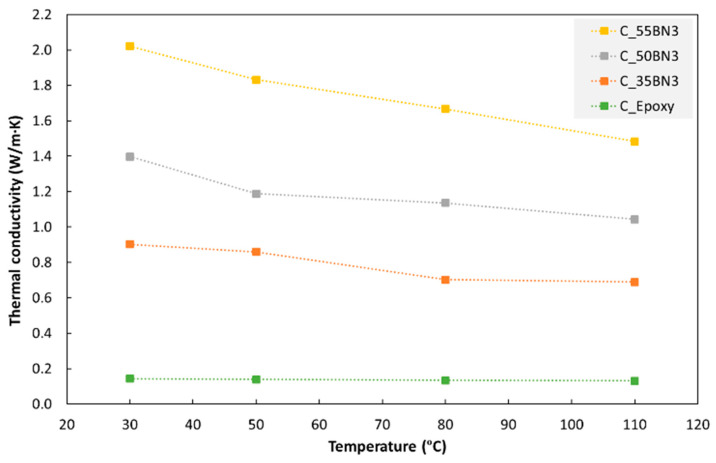
Thermal conductivity of cast composites with different hBN (BN3) fractions (0, 35, 50 and 55 wt%) as a function of temperature.

Figure 4 shows the thermal conductivity of cast hBN/epoxy composites as a function of the temperature. For all hBN fractions in the figure (0 to 55 wt%), the thermal conductivity decreases approximately linearly with increasing temperature. For pure epoxy, the conductivity drops by about 9% when increasing the temperature from 30 °C to 110 °C. For composites with 35–55 wt% hBN, the corresponding drop is about 25% (the differences between the three hBN fractions were not statistically significant). Similar temperature effects were observed for IM and PBF composites.

### 3.2. Hardness and Tensile Properties

**Figure 5:**
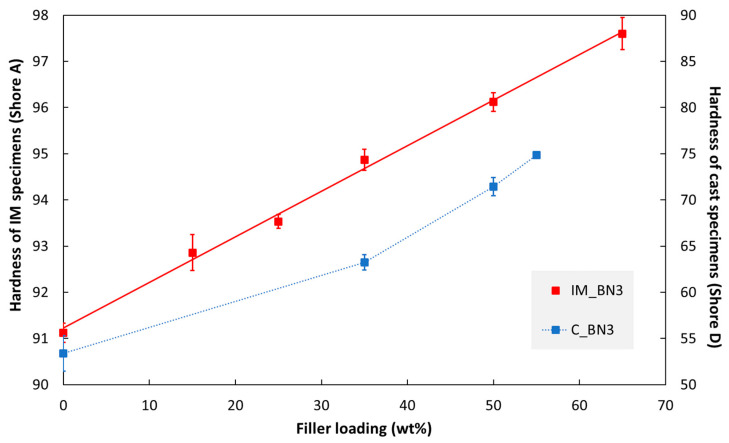
Hardness of injection moulded (IM) and cast (C) specimens as a function of hBN loading.

The hardness values of injection moulded and cast specimens are presented in Figure 5. In both cases, the hardness increases with increasing hBN content (note that two different Shore scales are used in the figure).

**Figure 6:**
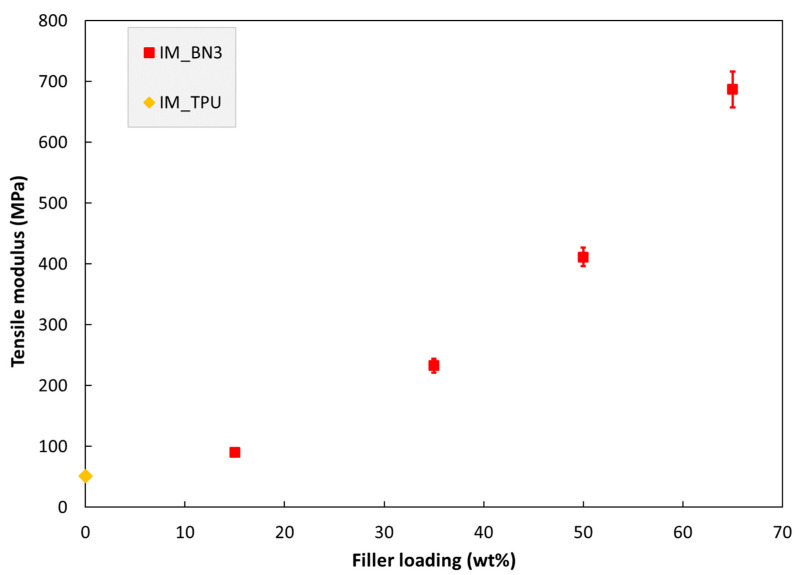
Tensile modulus of injection moulded specimens as a function of hBN loading. The specimen IM_TPU is 100% TPU.

**Figure 7:**
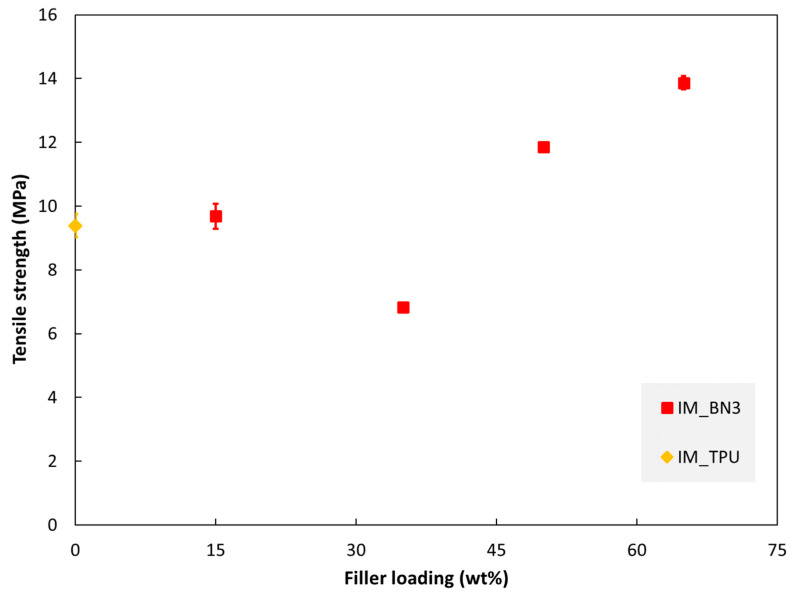
Tensile strength of injection moulded specimens as a function of hBN loading.

**Figure 8:**
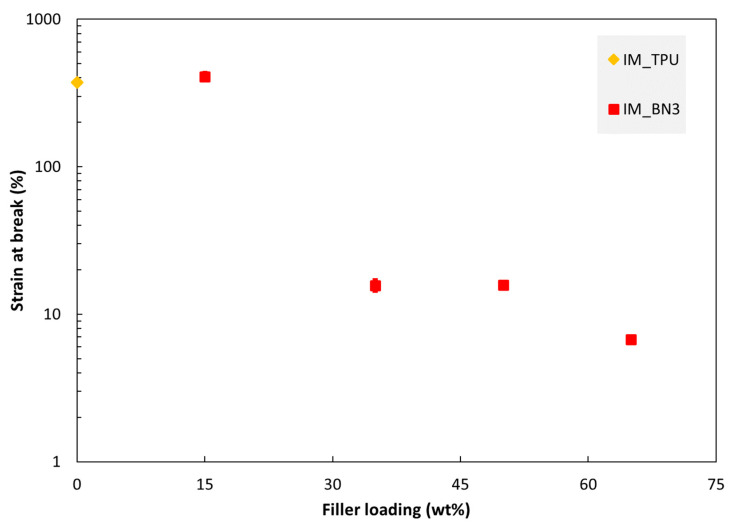
Strain at break of injection moulded specimens as a function of hBN loading.

The tensile properties of injection moulded specimens are shown in Figure 6, Figure 7 and Figure 8. The tensile modulus increases monotonously with increasing filler loading in this range. The strength and strain at break values are almost unaffected by adding 15% hBN. With 35% hBN, both these values are reduced. With 50% hBN, the strength values are higher than that for 100% TPU, while the strain at break values are similar to those for 35% hBN. With 65% hBN, the highest strength and the lowest strain at break are observed.

**Figure 9:**
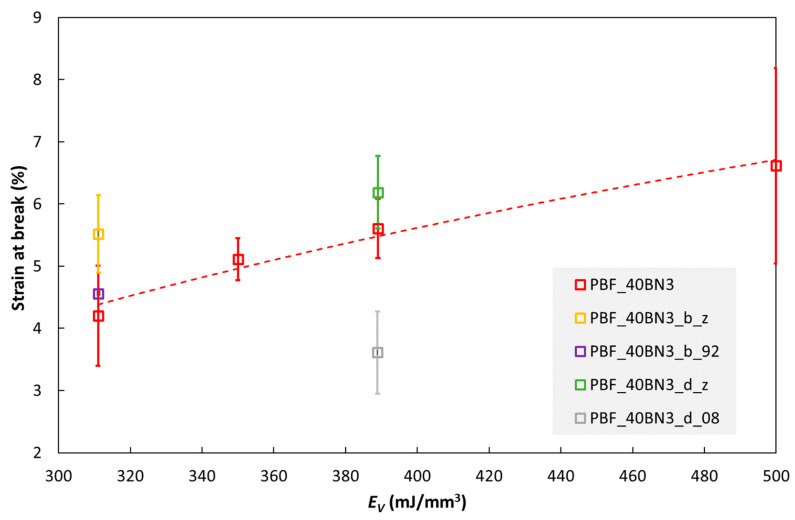
Strain at break of PBF specimens with 40 wt% hBN (BN3) as a function of volumetric energy density E_V_. The specimen codes are explained in Table S3 in the Supplementary Materials.

The PBF composites are more brittle than the IM composites, although pure TPU is also more brittle when processed by PBF. Figure 9 shows the strain at break of some PBF specimens as the function of printing parameters. Note the similarity with the trends for the thermal conductivity in Figure 3. For the main data series in this figure (PBF_40BN3; red open squares), the laser energy density, *E_V_*, was increased by increasing the laser power or decreasing the scanning speed. In these cases, the strain at break increases with increasing *E_V_*. However, increasing *E_V_* by reducing the layer thickness (*L*) from 0.1 to 0.08 mm reduces the strain at break, as seen for specimen PBF_40BN3_d_08. The two specimens built along the z direction (specimen names ending with “_z”) have higher strain at break than those printed in the XY plane (all the other data points in the figure).

### 3.3. hBN Platelet Orientation

#### Characterisation of hBN Platelet Orientation by XRD

**Figure 10:**
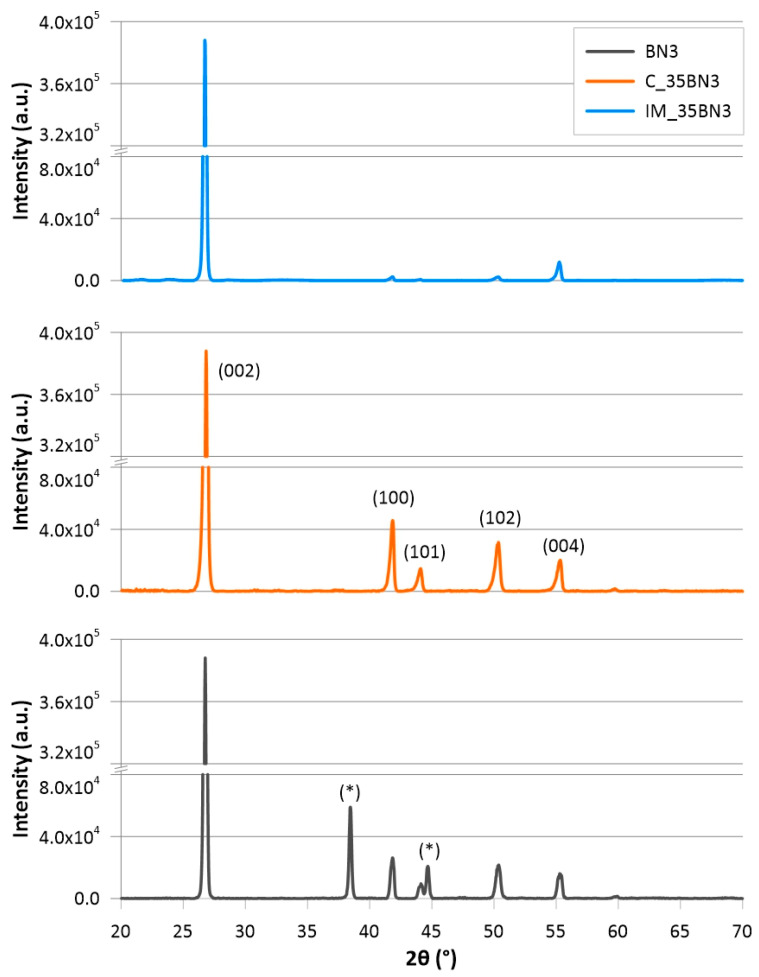
XRD patterns of pure BN (BN3) and specimens with 35 wt% BN3 fabricated by injection moulding (IM_35BN3) and casting (C_35BN3). The diffractogram of the pure hBN has peaks originating from the sample holder (marked with *) due to the small hBN sample size.

**Figure 11:**
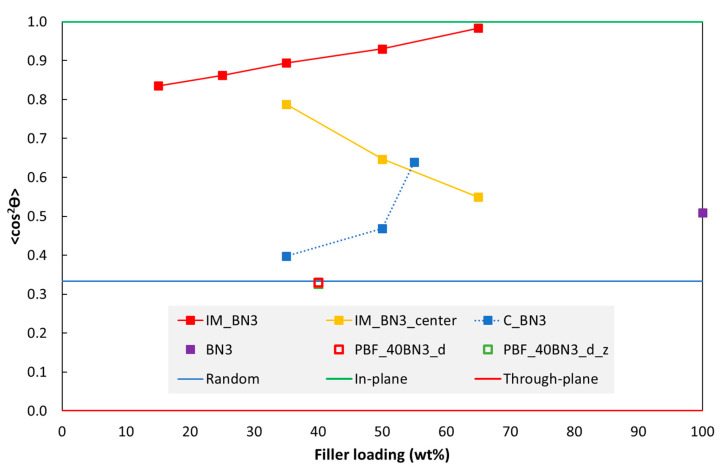
Orientation of hBN platelets ($\langle \cos^{2}\theta \rangle$ from Equation (3)) as a function of hBN loading in discs fabricated by injection moulding (IM_BN3), casting (C_BN3) and PBF (PBF_40BN3_d; PBF_40BN3_d_z). Data from the centre of an IM specimen (after grinding and polishing away half the thickness) are also included (IM_BN3_centre). The solid lines correspond to through-plane, random and in-plane orientations. The data points of the two PBF specimens overlap (open square symbols).

XRD was used to characterise the orientation of hBN platelets in the polymer matrices. Figure 10 shows an example of XRD data for specimens with 35 wt% BN3, fabricated by injection moulding and casting. The diffraction patterns of the two specimens have very different *100*/*002* peak ratios. Figure 11 shows the effect of hBN loading on the orientation.

## 4. Discussion

Section 4.1 starts with a discussion of the platelet orientation in injection moulded discs, focusing on the variation through the thickness and the effect of hBN loading. Then, the orientation in discs fabricated with the two other methods are discussed briefly. Section 4.2 discusses effects of hBN loading, hBN powder type and the processing method on the measured thermal conductivity, as well as the effect of test temperature. Section 4.3 compares our experimental conductivity data with the literature data. Section 4.4 compares our experimental conductivity data with model predictions. The mechanical properties are discussed in Section 4.5.

### 4.1. Orientation of hBN Platelets

Data for the orientation of hBN platelets in IM discs are shown in Figure 11. This $\langle \cos^{2}\theta \rangle$ value is dominated by diffraction from regions near the surface, typically the outer 0.3–0.4 mm, with gradually less intensity from deeper layers. This is based on the estimated X-ray penetration depths (see Experimental Section), and the XRD measurements with half-thickness discs (specimen IM_BN3_centre in Figure 11).

The $\langle \cos^{2}\theta \rangle$ values of hBN platelets near the surface of a “full” disc increases with increasing hBN loading, while the opposite trend is observed for “half-thickness” discs.

It can be questioned if the observed effect of hBN loading can be an artefact due to the X-ray absorption coefficient varying with the hBN loading. If we assume that the orientation is highest near the surface of the 2 mm disc and the linear X-ray absorption coefficient increases with BN loading (see Experimental Section), this could contribute to the observed trends; a higher BN loading would result in less diffracted intensity from deeper layers with lower orientation (or higher orientation for “half-thickness” discs), and, hence, a higher observed $\langle \cos^{2}\theta \rangle$ value as an artefact. It is difficult to quantify this effect because we do not know the orientation distribution through the cross section a priori. However, with the estimated low effect of hBN loading on the absorption coefficient, we believe that the effects of hBN loading on the $\langle \cos^{2}\theta \rangle$ values in Figure 11 reflect real effects in the IM specimens.

A $\langle \cos^{2}\theta \rangle$ value close to 1 means that hBN platelets are highly in-plane oriented. Our results, as shown in Figure 11, indicate that the centre region of the IM specimen (IM_BN3_centre) has lower $\langle \cos^{2}\theta \rangle$ values than the region near the surface. For the IM disc with 35 wt% hBN, the $\langle \cos^{2}\theta \rangle$ value of the half-specimen IM_BN3_centre is quite close to that of the full specimen IM_BN3, implying a more uniform orientation through the cross-section of the specimen.

A variation in platelet orientation through the disc thickness agrees with numerical simulations of platelet orientation induced by the injection moulding process (Figure S4 in the Supplementary Materials). A variation from the surface to the central region (core) is well known for IM parts, in particular for fibres [45], but also for plate-like particles [46]. The typical orientation resulting from the flow between two parallel mould cavity walls is a core-shell-skin structure with a shell layer of plates aligned along the walls (due to the high shear strain rate in this position) and a core layer with lower in-plane orientation, or even transverse orientation. Between the core and shell layers, there is a transition layer. The outer-most skin of a moulded part is deposited directly from the fountain flow at the flow front, and this skin layer typically has lower in-plane orientation than the shell layer.

As mentioned in Section S7 in the Supplementary Materials, the simulated maximum and minimum orientation through the thickness can be tuned by model parameter. The effect of hBN loading on increasing the maximum orientation through the thickness while reducing the minimum orientation value, as in Figure 11, can also be modelled qualitatively by decreasing the value of the particle–particle interaction parameter *C_I_* (Supplementary Materials). A similar experimental trend of the orientation in the shell layer increasing with particle loading was observed for glass fibres [45]. However, according to [47], there is no clear dependency of *C_I_* on the filler loading, and different experiments have showed contradictory results.

For cast discs of epoxy with hBN, the $\langle \cos^{2}\theta \rangle$ value also increases with increasing hBN loading (Figure 11), although the values are lower than for the IM discs. For an hBN loading of 35 wt%, $\langle \cos^{2}\theta \rangle$ is close to 1/3, implying random orientation. A random orientation would be expected due to the mixing prior to casting, and was also observed by Yuan et al. [4] and Lin et al. [21] in their reference specimens. The higher $\langle \cos^{2}\theta \rangle$ values (preferred in-plane orientation) for higher hBN loadings could be related to the high viscosity and the specimen preparation. Due to the high viscosity, the material had to be pressed into the open mould with a tool in order to make a specimen without voids. This could have induced an in-plane orientation of platelets, especially near the surfaces. A tendency for platelet stacking due to a high concentration [4,14] (less space for the platelets to arrange freely) could contribute to the orientation at a certain distance from the surface as well.

The two PBF discs with 40 wt% hBN in Figure 11, printed in the XY plane and “standing”, respectively, have a $\langle \cos^{2}\theta \rangle$ value close to 1/3, i.e., a random orientation. There are several studies of polymer PBF with added glass or carbon fibres, and these report a preferred fibre orientation in the powder spreading direction, i.e., the direction the powder recoater unit is travelling [48,49,50]. The degree of orientation depends on the type of recoater (roller, blade), the recoater speed and the layer thickness. The orientation mechanism is the shear flow induced by the recoater. Numerical models have been used to understand the flow dynamics in the recoating/packing process, and also the formation of voids [51]. In many cases, the fibre orientation is low. Hence, the nearly random orientation of the hBN platelets in our study is in line with the literature. The flow dynamics of the hBN platelets may also differ from that of the fibres in the literature. At the surface of the recoated layer, there may be some platelets being “combed down” to an in-plane (XY) orientation by the recoater. These oriented platelets, in combination with possible small voids/gaps between the fused layers, may have a negative effect on the thermal conductivity perpendicular to the layers, as well as on the mechanical properties.

### 4.2. Thermal Conductivity

The thermal conductivity increases with increasing hBN loading for all three processing methods in this study. The slope of the thermal conductivity vs. filler loading appears to increase at around 30 wt% filler (about 18 vol% for IM hBN/TPU), and with a further increase at loadings from about 50 wt% (about 34 vol% for IM hBN/TPU).

According to experimental literature on composites with hBN, the percolation threshold for thermal conductivity is about 20–25 vol% for randomly oriented platelets (studies with hBN size in the range of 3–8 µm [52], average particle size of 5 µm [21] and no size given [53]). For highly through-plane-orientated platelets, the threshold is reported to be about 10–15 vol% (studies with average particle sizes of 25 µm [54], 8 µm [55] and 5 µm [21]). Hence, the thermal percolation threshold depends on the orientation of the hBN platelets in the polymer matrix. Above the percolation threshold, the platelets form thermally conductive pathways in the polymer matrix. We will return to a discussion of conductivity vs. hBN fraction in the next section, when comparing experimental data with model predictions.

Injection moulded composites have lower conductivity than cast composites. This is due to the preferred in-plane orientation of the hBN platelets in the former case, as shown by the XRD data discussed in Section 4.1. Yuan et al. [4] also showed that a high in-plane orientation of the platelets leads to a low conductivity enhancement in the through-plane direction.

In a study of injection moulded polyamide 6 with Cu platelets, Heinle et al. [39] observed similar platelet orientations, and orientation variations through the specimen thickness, as in our study. Heinle et al. moulded specimens with different thicknesses (2, 3 and 4 mm), and the core/shell ratio increased with increasing specimen thickness (2, 3 and 4 mm). Due to a thicker core, 4 mm thick specimens had almost a three times higher through-plane conductivity than 2 mm thick specimens (for 40 vol% platelets). Heinle et al. also measured the conductivity in different directions; through-plane, in-plane along the flow direction and in-plane transverse to the flow. For 40 vol% platelets, the conductivities were almost the same in the two first directions, while it was almost two times higher in the third direction.

In addition to BN3, two other hBN types (BN1 and BN2) were used in some experiments (see Supplementary Materials, Sections S1 and S9). BN1 has smaller platelets than BN2 and BN3. BN2 mainly contains hBN platelets, while BN3 contains both platelets and spherical agglomerates of platelets (Figure S2a).

BN2 results in higher conductivity than BN3. Although some agglomerates are broken up during processing, the lower conductivity with BN3 is probably due to platelets being less dispersed. Powders with hBN agglomerates (as BN3) are claimed to have more isotropic properties and easier processing due to a lower viscosity from spherical fillers [5].

With the same hBN weight fraction in the composite, BN1 results in lower thermal conductivity than the two other powders. This effect is observed for both IM and PBF specimens, at all temperatures. The smaller platelets in BN1 probably results in a larger total surface area and smaller average distance between platelets in the composite (if randomly distributed). A larger surface area will reduce the conductivity of the composite if the matrix-filler interfacial thermal resistance is high [56]. On the other hand, above a certain platelet fraction, a reduction in the average distance between platelets can increase the conductivity due to a higher possibility of platelets coming into contact, thus leading to conducting “chains” of platelets and, consequently, percolation [57]. In our case, the negative effect of increased surface area seems to dominate. Different powders may also have varying tendencies for agglomeration and deagglomeration during processing, and this affects the conductivity. Li et al. [58] reported a similar size effect on thermal conductivity in polyimide films with micro- and nano-sized BN.

Note that the interfacial thermal resistance refers to the combined effect of two thermal resistances [59]: (1) the thermal contact resistance caused by poor mechanical and chemical bonding between the two phases and (2) the thermal boundary resistance due to differences in the physical properties of the two phases. In our case, the former resistance is probably dominating due to poor adhesion between the polymer matrix and the hBN surface.

The surface of the hBN particles can be modified chemically in order to improve the dispersion of hBN particles and achieve a stronger hBN/polymer interface, thereby improving the thermal conductivity and the mechanical properties [16,19,60,61,62]. There are wet and dry routes, including covalent methods (e.g., oxidation of hBN with a strong acid or base at high temperature and/or high pressure), non-covalent methods (e.g., coatings of organic compounds or inorganic particles) and solid-state methods (e.g., thermal treatment, high-energy radiation or strong mechanical forces) [16]. However, the chemical inertness and oxidation resistance of hBN make the functionalisation of hBN challenging.

In our study, chemical functionalisation was tried for the BN3 powder. The hBN particles were treated with a strong oxidation agent (nitric acid) followed by silanization (using the aminosilane coupling agent APTES) [19]. However, the coupling agent could not be attached to the hBN particles based on analyses using IR spectroscopy and energy dispersive X-ray spectroscopy.

The thermal conductivity of the polymer matrices and the hBN/polymer composites decreases as a function of the temperature (Figure 4). The relative decrease is more pronounced for the composites. This can be due to the mismatch in thermal expansion coefficients between the platelets (low expansion coefficient) and the polymer matrix (high expansion coefficient). When increasing the temperature, the mismatch will result in an increase in the distance between adjacent filler platelets in thermal pathways, and may also induce gaps between platelets and matrix. Both effects will reduce the conductivity of the composite [51,54].

### 4.3. Comparison with Published Thermal Conductivity Values for Composites with hBN

Although the main objective of this paper is to contribute to the understanding of thermal conductivity vs. hBN loading, hBN platelet orientation, etc., via processing method, it is also interesting to compare with published values for similar composites, as in Table 1. However, such comparisons are challenging, due to differences in mixing/processing methods, hBN loading, hBN particle size and matrix material.

- Compression moulded hBN/epoxy in ref. [7] achieved a thermal conductivity of 7 W/m·K with 95 wt% hBN. Cast hBN/epoxy in ref. [8] reached a thermal conductivity of 5.3 W/m·K with 57 vol% hBN (with size of 5–11 μm), but values for lower hBN loadings were not presented. Our highest thermal conductivity for cast hBN/epoxy composites was 2.0 W/m·K for 55 wt% (about 28 vol%) hBN (with size of ca. 20 μm). Our casting resin could not be processed with a filler loading higher than this.
- For injection moulding, ref. [10] achieved 3.7 W/m·K for PE with 50 vol% hBN with particle diameter 4–5 μm. Our injection moulded composite based on TPU with 65 wt% hBN (about 48 vol%) reached a thermal conductivity of 2.1 W/m·K. The higher value in ref. [10] could be due to a better dispersion and more homogeneous distribution of hBN particles.
- For PBF composites, refs. [12,13] achieved relatively higher thermal conductivities than in our study. This could be due to synergetic effects of using two filler types (hBN and Al_2_O_3_ in ref. [12], hBN and AlN in ref. [13]).
- For PBF composites with only one filler type, our results are similar to those in ref. [11] with regard to (hBN/PA12). Ref. [11] reported a thermal conductivity of 0.55 W/m·K with 40 wt% hBN (275% higher than the pure PA12 processed with PBF). Our best PBF composite with 40 wt% hBN had a thermal conductivity of 0.56 W/m·K (ca. 460% higher than the pure TPU processed with PBF).

Hence, hBN particle type and mixing method are important factors, as well as surface treatment of the particles. The matrix material, in particular its viscosity, must be carefully selected in order to obtain processable composites with higher particle loadings. Furthermore, synergetic effects can be achieved when using two particle types.

### 4.4. Experimental Thermal Conductivity vs. Model Predictions

Comparing the experimental thermal conductivity data with models can aid the discussion. Many models have been developed for the thermal conductivity of composites. This section presents data obtained with four models (see Supplementary Materials for details and also some comparisons). One reason for including these four models is that they provide quite different predictions. Many articles only use one of these models without arguing for their choice of model. Furthermore, one of the models (the Sun model) is quite new, and, to the best of our knowledge, we are the first to use and evaluate it (apart from the cited article by Sun et al.).

#### 4.4.1. The Thermal Conductivity Model of Nan et al.

Nan et al. [36] introduced a Maxwell–Garnett-type effective medium approximation (EMA) model (see Section S11 in the Supplementary Materials) which has been used in many studies of hBN/polymer composites [4,20,53]. The Nan model includes effects of particle shape (ellipsoids, which can represent platelets), particle orientation and interfacial thermal resistance. However, it is limited to particles with isotropic thermal conductivity, while hBN platelets are anisotropic. Furthermore, this model does not take into account filler-filler contact or nonuniform particle distributions (nor do any of the models in this section). Hence, it is restricted to low filler fractions, and it cannot predict the percolation threshold, or effects of non-homogeneous platelet distributions or poor platelet dispersion (stacks and agglomerates).

Some insight can be gained by comparing our experimental data with the Nan model, with different values for the filler-matrix interfacial thermal resistance (*R_BD_*) and platelet orientation ($\langle \cos^{2}\theta \rangle$) in the model. Section S11 in the Supplementary Materials provides details about the Nan model and a background for the model parameters used in our study, including a simple sensitivity analysis.

**Figure 12:**
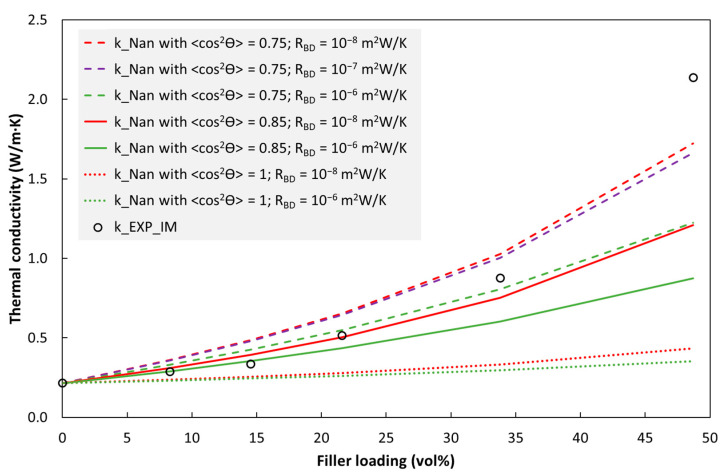
Nan model compared with the measured values for injection moulded BN3/TPU (k_EXP_IM). Model parameters are given in the legend and in the Supplementary Materials. For all $\langle \cos^{2}\theta \rangle$ values in this figure, the curves for R_BD_ = 0 and 10^−8^ m^2^W/K almost overlap.

For the injection moulded discs, a simplified picture is that the average platelet orientation through the disc thickness is almost independent of hBN loading, with $\langle \cos^{2}\theta \rangle$ of around 0.8, referring to the average of red and yellow solid square symbols in Figure 11. Figure 12 compares experimental data with the Nan model with relevant model parameters. For hBN loadings of up to about 15 vol%, the experimental data are merely below the model predictions (almost on the green solid line corresponding to $\langle \cos^{2}\theta \rangle$ = 0.85 and *R_BD_* = 10^−6^ m^2^W/K). This is reasonable; even though the experimental $\langle \cos^{2}\theta \rangle$ (average over the thickness) may be comparatively lower than 0.85, the *R_BD_* value may be lower than 10^−6^ m^2^W/K, and the platelets are probably not homogeneously distributed and not perfectly dispersed (from agglomerates).

The steeper slope for the experimental data in the range 15–35 vol% is probably due to the formation of some platelet-platelet contact, resulting in local conductive paths and a boost in conductivity. This could be represented as a reduction in the effective *R_BD_* value for the Nan model (here, “effective” means that there is some filler-filler contact in the specimens which is not accounted for in the model, in which *R_BD_* is strictly defined to represent filler-matrix contact). Other causes, however less likely, could be that there is a substantial core in the disc with a lower $\langle \cos^{2}\theta \rangle$ than which was captured by our measurements, or that the filler-matrix resistance decreases with increasing hBN loading.

The last experimental data point at that 48 vol% is above the Nan model predictions with reasonable input parameters. The Nan model predicts this datapoint if $\langle \cos^{2}\theta \rangle$ is set to 0.67 and *R_BD_* is set to 0. However, the $\langle \cos^{2}\theta \rangle$ value is probably higher than 0.67, and the mismatch between the model and the experimental data is probably due to even more platelet-platelet contact in the specimen (than at lower loadings). Finally, note that BN3 data were used in Figure 12 while hBN-type BN2 resulted in even higher conductivity (Figure S7 in the Supplementary Materials), while having roughly the same platelet aspect ratio.

A similar trend is observed for the experimental data of cast specimens vs. the Nan model, see Figure S13 in Section S12 in the Supplementary Materials. Hence, also in the cast specimens, platelet-platelet contact seems to form at high hBN loadings.

#### 4.4.2. The Thermal Conductivity Model of Ordóñez-Miranda et al.

Ordóñez-Miranda et al. [37] combined the Nan model above with the Bruggeman integration principle. The resulting model is claimed to be better at high particle volume fractions than the Nan model. However, Ordóñez-Miranda et al. only considered the case with a random orientation of particles.

As shown in Figure S14 in the Supplementary, the Ordóñez-Miranda model predictions are quite different from those of the Nan model (Figure S13). When comparing with the experimental data for cast specimens, and assuming a random orientation of platelets, the Ordóñez-Miranda model is closer to the experimental values than the Nan model when using relevant values for the interfacial thermal resistance. Furthermore, with the Ordóñez-Miranda model, only a very small reduction in interfacial thermal resistance (corresponding to the formation of some chains of platelets effective in the through-plane direction) is needed to capture the last data point (with an hBN volume fraction of 38.5%). The Nan model with random orientation cannot capture this data point, even with zero interfacial thermal resistance.

#### 4.4.3. The Thermal Conductivity Model of Sun et al.

Based on a finite element model, Sun et al. [38] derived an analytical model for the thermal conductivity of hBN/polymer composites. In addition to the effects included in the Nan model above, the Sun model also takes into account the anisotropic thermal conductivity of the hBN platelets. Hence, in principle, it should be more accurate than the Nan model. However, the Sun model has the same limitations as the models above regarding an absence of filler-filler contact and a homogeneous platelet distribution. The Sun model and our implementation are presented in Section S13 of the Supplementary Materials.

The Sun model generally predicts lower conductivities than the Nan model. For the cast specimens, the Sun model underestimates the conductivity for all three data points in the interval 20–40 vol% hBN, see Figure S15 in the Supplementary Materials. As for the Nan model, the experimental data has a larger curvature vs. filler loading (vol% hBN) than the model predictions.

#### 4.4.4. The Lewis-Nielsen Thermal Conductivity Model

This simple model has been used in studies of composites with platelets [39,63]. In addition to the conductivities of the two phases, the model has two semi-empirical constants (see Section S14 in the Supplementary Materials for details). The first constant represents the maximum volume fraction (often 0.8–0.85 for platelets [39,63]), and the second is a geometry factor for the particles. This geometry factor represents the effective particle shape in the direction in which the conductivity is measured. Hence, the average particle orientation is embedded in this geometry factor. The drawback of this model is that the geometry factor is mainly an empirical fitting parameter. Some comparisons between model predictions and experimental data are shown in Section S14 in the Supplementary Materials.

Heinle et al. [39] used a three-layer Lewis-Nielsen model for the through-plane thermal conductivity of injection moulded specimens. The three layers through the thickness of the specimens represented the two symmetric shell layers (with high in-plane orientation of Cu platelets) and the core layer (with lower orientation). This model resulted in good predictions of thermal conductivity vs. the Cu platelet volume fraction for different specimen thicknesses (2, 3 and 4 mm), which had different shell/core ratios. The model also predicted the conductivity in different directions (through-plane, in-plane along the flow direction and in-plane transverse to the flow).

### 4.5. Hardness and Tensile Properties

The high stiffness and strength of hBN platelets can, in principle, enhance the hardness, stiffness and strength of thermoplastics. However, the interphase between the two is weak [5], thus reducing the effectiveness of the platelets and rather inducing defects which reduce mechanical properties, in particular those involving strains above the elastic limit, i.e., most properties except the modulus of the composite. Prindl [64] injection moulded three thermoplastic materials (a polypropylene, a polyamide 6 and a thermoplastic elastomer) with hBN and found that properties such as strain at break, tensile strength and impact strength generally decreased when adding hBN. The least negative effect was observed for the polyamide 6.

The hardness enhancement effect of hBN platelets is about the same in injection moulded and cast specimens (Figure 5). The slightly larger relative enhancement in the former case could be related to the platelet concentration either in the outer layer or the platelet orientation.

The relative modulus enhancement (Figure 6) is larger than the relative hardness enhancement. This was expected due to the lower strain level for the modulus determination. Nevertheless, the modulus enhancement is rather low, given the high modulus of the hBN platelets relative to that of the TPU.

Regarding tensile strength and strain at break vs. hBN fraction (Figure 7 and Figure 8), the trends above 15% hBN could be explained by two mechanisms which occur when adding fillers. Increasing the hBN content reduces the ductility, i.e., reduces the strain at break. However, increasing the hBN content also reinforces the material, i.e., shifts the stress–strain curve upwards (to a higher stress for a given strain). From 15 to 35% hBN, the first mechanism dominates both the strength and strain at break, while from 35 to 50%, the second mechanism dominates.

In addition to BN3, another hBN type (BN2) was also used in some tests (material data and results are shown in Sections S1, S5 and S10 in the Supplementary Materials). BN2 mainly contains hBN platelets, while BN3 contains both hBN platelets and spherical agglomerates of hBN platelets (Figure S2a). There was no clear effect of hBN type on the tensile modulus. This could be due to the low strain, for which effects of particle size may be small. For the strength and strain at break, there are some effects of hBN type that are statistically significant. Especially at 35% hBN, but also at 50% hBN, BN2 results in a higher strength than BN3. This could be due to the agglomerates in BN3. At 50% hBN, the higher strength with BN2 could partly be due to better particle dispersion (and no agglomerates) and less micro-voids and imperfections. This agrees with the fact that BN2 also resulted in the highest thermal conductivity at this loading (see Figure S7 in the Supplementary Materials).

The PBF composites are more brittle than the IM composites due to voids caused by the thermal history and absence of melt flow in the PBF process (the flow in the IM process may relatively improve the wetting of hBN particles with TPU melt). In addition, the type of recoater used may not be optimal for such composites, see also Section 4.1. A decrease in tensile properties when adding hBN was also observed in the PBF study by Yang et al. [11], although they used hBN/PA12 co-powders, prepared by solid-state shear milling and cryogenic pulverisation.

Increasing the energy density in the PBF process improves the tensile strength and strain at break to a certain degree (Figure 9) due to better melting and fusion. However, the energies are high and there is a risk for the chemical degradation of the TPU. It may be that the hBN platelets reflect some of the laser energy, and they will also spread the heat away from the area hit by the laser beam.

The PBF specimens built along the z direction have higher strength and higher strain at break (and also higher thermal conductivity) than those printed in the XY plane. Usually, the opposite is observed for PBF, whereby specimens printed in the z direction have lower strength and strain at break. In our case, it could be that the bulk temperatures in the part during printing are higher for parts printed in the z direction. A difference in temperature history could mean a difference in achieving some TPU-hBN adhesion and reducing voids, and this could dominate over the usual “weakest chain” interlayer failure of PBF parts.

## 5. Conclusions

This study has provided new insights on the effects of the fabrication method and hBN loading on the thermal conductivity and mechanical properties of hBN/polymer composites. Injection moulding, casting and powder bed fusion were utilised for fabricating composite specimens. The hBN platelet orientation in the specimens was characterised by XRD measurements.

Injection moulding induced a preferred orientation of the platelets, with the platelet normal along the thickness direction of the specimens. Furthermore, the orientation varied through the thickness of the moulded specimens, and it increased with increasing hBN fraction. The platelet orientation in injection moulded specimens agreed qualitatively with numerical simulations. Casting only resulted in a low preferred orientation, and powder bed fusion resulted in an almost random orientation.

With the process equipment used, the maximum practical hBN loading was 65 wt% for injection moulding with TPU as matrix, 55 wt% for casting with epoxy and 40 wt% for powder bed fusion with TPU. The thermal conductivity of the composites increased with increasing hBN loading. The highest thermal conductivity in this study (2.1 W/mK) was obtained with injection moulding with 65 wt% hBN. However, for a given hBN loading, casting resulted in a higher thermal conductivity than the other two methods. This was partly due to the platelet orientation in cast specimens (in particular being more favourable than the orientation induced by injection moulding) and partly due to the matrix material (the epoxy probably resulting in a relatively lower interfacial thermal resistance, and also less porosity).

The conductivity and orientation data were discussed by a comprehensive comparison with four models for the thermal conductivity of composites. At low hBN loadings, the Nan model resulted in a fair prediction of the conductivity. Underprediction at higher loading is interpreted to be due to the fact that this model does not take into account the formation of platelet-platelet contact, resulting in local conducting paths (pre-percolation). The Ordóñez-Miranda model is limited to a random platelet orientation, but it predicts the experimental data for cast specimens better than the Nan model. Hence, the Ordóñez-Miranda model should be generalised to account for particle orientation.

Adding hBN increased the hardness and tensile modulus of the materials. For injection moulded specimens, the tensile strength had a minimum vs. hBN loading, while the strain at break decreased monotonously, with a sharp reduction between 15 and 35% hBN. These trends can be explained by two mechanisms which occur when adding hBN: reinforcement and embrittlement. Powder bed fusion resulted in even more brittle composites, and some type of hBN-polymer compatibilization is probably needed to achieve adequate mechanical properties, in addition to process enhancements.

## Figures and Tables

**Table 1:** Some studies of hBN/polymer composites. (wt% = weight percentage, vol% = volume percentage).

Materials	Filler Loading	Processing Method	Thermal Conductivity
hBN/epoxy [7]	95 wt%	Compression moulding	21.3 W/m·K (in-plane) 7 W/m·K (through-plane)
hBN/epoxy [8]	57 vol%	Casting	5.27 W/m·K (through-plane)
hBN/polyimide [9]	60 vol%	Spin-cast (film)	17.5 W/m·K (in-plane) 5.4 W/m·K (through-plane)
hBN/PE [10]	50 vol%	Injection moulding	3.66 W/m·K
hBN/PA12 [11]	40 wt%	Powder bed fusion	0.55 W/m·K (77% higher than pure PA12)
hBN/Al_2_O_3_/PA12 [12]	15 wt% hBN and 35 wt% Al_2_O_3_	Powder bed fusion	1.05 W/m·K (275% higher than pure PA12)
hBN/AlN/TPU [13]	15 wt% hBN and 20 wt% AlN	Powder bed fusion	0.9 W/m·K (391% higher than pure TPU)
hBN/TPU [14]	30 wt%	Fused deposition modelling (material extrusion)	1.51 W/m·K (in-plane) 1.26 W/m·K (through-plane)

**Table 2:** Materials used for preparation of hBN/polymer composites.

Short Name	Description	Product Name, Supplier
BN3 ^a^	hBN powder. Platelet agglomerates with D_50_ of 20 µm and size distribution in the range (0.5–31) µm, BET ~4 m^2^/g	HeBoFill CL-ADH 020, Henze Boron Nitride Products AG, Lauben, Germany
TPU	Thermoplastic polyurethane (in the form of powder). An elastomer with Shore A hardness 88.	Ultrasint TPU 88A, BASF, Ludwigshafen am Rhein, Germany
Epoxy ^b^	An epoxy system (for casting) containing: - 35 wt% unmodified bisphenol-F epoxy resin (Araldite GY 285-1) - 35 wt% reactive diluent (Araldite DY 026) - 30 wt% amine-based curing agent (Jeffamine D-230 Polyetheramine)	Huntsman, The Woodlands, TX, USA

^a^ Information regarding BN3 as provided by the supplier [40]. Size distributions of BN3 are shown in Figure S1 in the Supplementary Materials. The BN3 powder had a partly agglomerated particle structure, claimed to provide good lubricating properties and low viscosity increase [40]. Platelets and spherical agglomerates are shown in Figure S2a in the Supplementary Materials. ^b^ The epoxy system was formulated to have a low viscosity, suitable for preparing composites with high filler content.

## Data Availability

The raw/processed data required to reproduce these findings cannot be shared at this time due to legal restrictions.

## Supplementary Materials

The following supporting information can be downloaded at: https://www.mdpi.com/article/10.3390/polym15061552/s1. The Supplementary Materials contain additional details about materials (Sections S1 and S3) and experiments (Sections S2, S4–S6), as well as additional results on numerical simulation of platelet orientation in injection moulded discs (Section S7); DSC measurement (Section S8); additional data for thermal conductivity and tensile properties with some other hBN types (Sections S9 and S10); and thermal conductivity models vs. experimental data (Sections S11–S14). References [4,5,20,36,37,38,39,40,53,55,65,66,67] are cited in the Supplementary Materials.

## References

[1] Tummala R. (2001). Fundamentals of Microsystems Packaging.

[2] Bhanushali S., Ghosh P., Simon G., Cheng W. (2017). Copper Nanowire-Filled Soft Elastomer Composites for Applications as Thermal Interface Materials. Adv. Mater. Interfaces.

[3] Bahru R., Hamzah A., Mohamed M.A. (2020). Thermal management of wearable and implantable electronic healthcare devices: Perspective and measurement approach. Int. J. Energy Res..

[4] Yuan C., Duan B., Li L., Xie B., Huang M., Luo X. (2015). Thermal Conductivity of Polymer-Based Composites with Magnetic Aligned Hexagonal Boron Nitride Platelets. ACS Appl. Mater. Interfaces.

[5] Chen H., Ginzburg V., Yang J., Yang Y., Liu W., Huang Y., Du L., Chen B. (2016). Thermal conductivity of polymer-based composites: Fundamentals and applications. Prog. Polym. Sci..

[6] Li D., Zeng D., Chen Q., Wei M., Song L., Xiao C., Pan D. (2019). Effect of different size complex fillers on thermal conductivity of PA6 thermal composites. Plast. Rubber Compos..

[7] Zhu Z., Wang P., Lv P., Xu T., Zheng J., Ma C., Yu K., Feng W., Wei W., Chen L. (2018). Densely packed polymer/boron nitride composite for superior anisotropic thermal conductivity. Polym. Compos..

[8] Xu Y., Chung D.D.L. (2000). Increasing the thermal conductivity of boron nitride and aluminum nitride particle epoxy-matrix composites by particle surface treatments. Compos. Interfaces.

[9] Tanimoto M., Yamagata T., Miyata K., Ando S. (2013). Anisotropic thermal diffusivity of hexagonal boron nitride-filled polyimide films: Effects of filler particle size, aggregation, orientation, and polymer chain rigidity. ACS Appl. Mater. Interfaces.

[10] Lee G.-W., Park M., Kim J., Lee J., Yoon H.G. (2006). Enhanced thermal conductivity of polymer composites filled with hybrid filler. Compos. Part A Appl. Sci. Manuf..

[11] Yang L., Wang L., Chen Y. (2020). Solid-state shear milling method to prepare PA12/boron nitride thermal conductive composite powders and their selective laser sintering 3D-printing. J. Appl. Polym. Sci..

[12] Yuan Y., Wu W., Hu H., Liu D., Shen H., Wang Z. (2021). The combination of Al_2_O_3_ and BN for enhancing the thermal conductivity of PA12 composites prepared by selective laser sintering. RSC Adv..

[13] Zhang X., Wu W., Zhao T., Li J. (2022). The combination of AlN and h-BN for enhancing the thermal conductivity of thermoplastic polyurethane composites prepared by selective laser sintering. J. Appl. Polym. Sci..

[14] Gao J., Hao M., Wang Y., Kong X., Yang B., Wang R., Lu Y., Zhang L., Gong M., Zhang L. (2022). 3D printing boron nitride nanosheets filled thermoplastic polyurethane composites with enhanced mechanical and thermal conductive properties. Addit. Manuf..

[15] Yu C., Zhang J., Tian W., Fan X., Yao Y. (2018). Polymer composites based on hexagonal boron nitride and their application in thermally conductive composites. RSC Adv..

[16] Zheng Z., Cox M., Li B. (2018). Surface modification of hexagonal boron nitride nanomaterials: A review. J. Mater. Sci..

[17] Zhang Y., Gao W., Li Y., Zhao D., Yin H. (2019). Hybrid fillers of hexagonal and cubic boron nitride in epoxy composites for thermal management applications. RSC Adv..

[18] Merlo A., Mokkapati V., Pandit S., Mijakovic I. (2018). Boron nitride nanomaterials: Biocompatibility and bio-applications. Biomater. Sci..

[19] Ciofani G., Genchi G., Liakos I., Athanassiou A., Dinucci D., Chiellini F., Mattoli V. (2012). A simple approach to covalent functionalization of boron nitride nanotubes. J. Colloid Interface Sci..

[20] Xue Y., Li X., Wang H., Zhao F., Zhang D., Chen Y. (2019). Improvement in thermal conductivity of through-plane aligned boron nitride/silicone rubber composites. Mater. Des..

[21] Lin Z., Liu Y., Raghavan S., Moon K., Sitaraman S., Wong C.P. (2013). Magnetic alignment of hexagonal boron nitride platelets in polymer matrix: Toward high performance anisotropic polymer composites for electronic encapsulation. ACS Appl. Mater. Interfaces.

[22] Tebeta R.T., Fattahi A., Ahmed N.A. (2020). Experimental and numerical study on HDPE/SWCNT nanocomposite elastic properties considering the processing techniques effect. Microsyst. Technol..

[23] Crawford R.J. (1998). Plastics Engineering.

[24] Ligon S.C., Liska R., Stampfl J., Gurr M., Mülhaupt R. (2017). Polymers for 3D Printing and Customized Additive Manufacturing. Chem. Rev..

[25] Yuan S. (2018). Development and Optimization of Selective Laser Sintered-Composites and Structures for Functional Applications. Ph.D. Thesis.

[26] Yuan S., Shen F., Chua C., Zhou K. (2019). Polymeric composites for powder-based additive manufacturing: Materials and applications. Prog. Polym. Sci..

[27] Sillani F., de Gasparo F., Schmid M., Wegener K. (2021). Influence of packing density and fillers on thermal conductivity of polymer powders for additive manufacturing. Int. J. Adv. Manuf. Technol..

[28] Lupone F., Padovano E., Ostrovskaya O., Russo A., Badini C. (2021). Innovative approach to the development of conductive hybrid composites for Selective Laser Sintering. Compos. Part A Appl. Sci. Manuf..

[29] Yuan S., Zheng Y., Chua C., Yan Q., Zhou K. (2018). Electrical and thermal conductivities of MWCNT/polymer composites fabricated by selective laser sintering. Compos. Part A Appl. Sci. Manuf..

[30] Ronca A., Rollo G., Cerruti P., Fei G., Gan X., Buonocore G., Lavorgna M., Xia H., Silvestre C., Ambrosio L. (2019). Selective Laser Sintering Fabricated Thermoplastic Polyurethane/Graphene Cellular Structures with Tailorable Properties and High Strain Sensitivity. Appl. Sci..

[31] Lanzl L., Wudy K., Greiner S., Drummer D. (2019). Selective laser sintering of copper filled polyamide 12: Characterization of powder properties and process behavior. Polym. Compos..

[32] Gruber P., Ziółkowski G., Olejarczyk M., Grochowska E., Hoppe V., Szymczyk-Ziółkowska P., Kurzynowski T. (2022). Influence of bioactive metal fillers on microstructural homogeneity of PA12 composites produced by polymer Laser Sintering. Arch. Civ. Mech. Eng..

[33] Hon K.K.B., Gill T.J. (2003). Selective Laser Sintering of SiC/Polyamide Composites. CIRP Annals..

[34] Do N.B.D., Andreassen E., Edwardsen S., Lifjeld A., Aasmundtveit K., Nguyen H.-V., Imenes K. (2021). Thermal management of an interventional medical device with double layer encapsulation. Exp. Heat Transf..

[35] Frick A., Rochman A. (2004). Characterization of TPU-elastomers by thermal analysis (DSC). Polym. Test..

[36] Nan C.W., Birringer R., Clarke D., Gleiter H. (1997). Effective thermal conductivity of particulate composites with interfacial thermal resistance. J. Appl. Phys..

[37] Ordóñez-Miranda J., Alvarado-Gil J., Medina-Ezquivel R. (2010). Generalized Bruggeman Formula for the Effective Thermal Conductivity of Particulate Composites with an Interface Layer. Int. J. Thermophys..

[38] Sun Y., Zhou L., Han Y., Cui L., Chen L. (2020). A new anisotropic thermal conductivity equation for h-BN/polymer composites using finite element analysis. Int. J. Heat Mass Transf..

[39] Heinle C., Brocka Z., Hülder G., Ehrenstein G., Osswald T. Thermal conductivity of polymers filled with non-isometric fillers: A process dependent, anisotropic property. Proceedings of the 67th Annual Technical Conference of the Society of Plastics Engineers (ANTEC 2009, Chicago).

[40] Henze Boron Nitride Products AG (2020). HeBoFill ® CL-ADH 020. Technical Datasheet.

[41] Zajas J., Heiselberg P. (2013). Measurements of thermal diffusivity, specific heat capacity and thermal conductivity with LFA 447 apparatus. Aalb. Univ..

[42] Salmon D.R., Brandt R., Tye R.P. (2010). Pyroceram 9606, A certified ceramic reference material for high-temperature thermal transport properties: Part 2-certification measurements. Int. J. Thermophys..

[43] Polymer Orientation. http://www.personal.psu.edu/irh1/PDF/Orientation.pdf.

[44] Girgsdies F. (2015). Peak Profile Analysis in X-ray Powder Diffraction.

[45] Jørgensen J.K., Andreassen E., Salaberger D. (2019). The effect of fiber concentration on fiber orientation in injection molded film gated rectangular plates. Polym. Compos..

[46] Granlund H., Andreassen E., Skjønsfjell E., Høydalsvik K., Diaz A., Breiby D.W. (2014). Measuring and simulating morphology gradients in injection-molded talc-reinforced isotactic polypropylene. J. Polym. Sci. B Polym. Phys..

[47] Kugler S.K., Kech A., Cruz C., Osswald T. (2020). Fiber Orientation Predictions—A Review of Existing Models. J. Compos. Sci..

[48] Chen H., Zhu W., Tang H., Yan W. (2021). Oriented structure of short fiber reinforced polymer composites processed by selective laser sintering: The role of powder-spreading process. Int. J. Mach. Tools Manuf..

[49] Khudiakova A., Berer M., Niedermair S., Plank B., Truszkiewicz E., Meier G., Stepanovsky H., Wolfahrt M., Pinter G., Lackner J. (2020). Systematic analysis of the mechanical anisotropy of fibre-reinforced polymer specimens produced by laser sintering. Addit. Manuf..

[50] Heckner T., Seitz M., Raisch S., Huelder G., Middendorf P. (2020). Selective Laser Sintering of PA6: Effect of Powder Recoating on Fibre Orientation. J. Compos. Sci..

[51] Tan P., Shen F., Tey W., Zhou K. (2021). A numerical study on the packing quality of fibre/polymer composite powder for powder bed fusion additive manufacturing. Virtual Phys Prototyp..

[52] Kargar F., Barani Z., Salgado R., Debnath B., Lewis J., Aytan E., Lake R., Balandin A.A. (2018). Thermal Percolation Threshold and Thermal Properties of Composites with High Loading of Graphene and Boron Nitride Fillers. ACS Appl. Mater. Interfaces.

[53] Pan C., Zhang J., Kou K., Zhang Y., Wu G. (2018). Investigation of the through-plane thermal conductivity of polymer composites with in-plane oriented hexagonal boron nitride. Int. J. Heat Mass Transf..

[54] Ding D., Zou M., Wang X., Qin G., Zhang S., Chan S., Meng Q., Liu Z., Zhang Q., Chen Y. (2022). Thermal conductivity of polydisperse hexagonal BN/polyimide composites: Iterative EMT model and machine learning based on first principles investigation. Chem. Eng. J..

[55] Liu J., Li W., Guo Y., Zhang H., Zhang Z. (2019). Improved thermal conductivity of thermoplastic polyurethane via aligned boron nitride platelets assisted by 3D printing. Compos. Part A Appl. Sci. Manuf..

[56] Ordonez-Miranda J., Alvarado-Gil J.J. (2012). Thermal conductivity of nanocomposites with high volume fractions of particles. Compos. Sci. Technol..

[57] Jing X., Zhao W., Lan L. (2000). The effect of particle size on electric conducting percolation threshold in polymer/conducting particle composites. J. Mater. Sci. Lett..

[58] Li T.-L., Hsu S.L.-C. (2010). Enhanced Thermal Conductivity of Polyimide Films via a Hybrid of Micro- and Nano-Sized Boron Nitride. J. Phys. Chem. B.

[59] Pietrak K., Wiśniewski T. (2014). A review of models for effective thermal conductivity of composite materials. J. Power Technol..

[60] Daneshmehr S., Román F., Hutchinson J.M. (2019). The surface modification of boron nitride particles. J. Therm. Anal. Calorim..

[61] Cui Z., Oyer A., Glover A., Schniepp H., Adamson D.H. (2014). Large scale thermal exfoliation and functionalization of boron nitride. Small.

[62] Ryu S., Kim K., Kim J. (2018). Silane surface treatment of boron nitride to improve the thermal conductivity of polyethylene naphthalate requiring high temperature molding. Polym. Compos..

[63] Hill R.F., Supancic P.H. (2004). Thermal Conductivity of Platelet-Filled Polymer Composites. J. Am. Ceram. Soc..

[64] Prindl J. (2015). Enhancing Thermal Conductivity of Hexagonal Boron Nitride Filled Thermoplastics for Thermal Interface Management. Master’s Thesis.

[65] Henze Boron Nitride Products AG (2020). HeBoFill ® LL-SP 120. Technical Datasheet.

[66] Tseng H.-C., Chang R.-Y., Hsu C.-H. (2020). Comparison of recent fiber orientation models in injection molding simulation of fiber-reinforced composites. J. Thermoplast. Compos. Mater..

[67] Mazahir S.M., Velez-Garcia G.M., Wapperom P., Baird D. (2015). Fiber orientation in the frontal region of a center-gated disk: Experiments and simulation. J. Nonnewton Fluid Mech..

