# Supplementary material for: Thermal Conductivity and Mechanical Properties of Polymer Composites with Hexagonal Boron Nitride—A Comparison of Three Processing Methods: Injection Moulding, Powder Bed Fusion and Casting
Source: Polymers (Basel). 2023 Mar 21;15(6):1552. doi: 10.3390/polym15061552 (PMC10053309; doi:10.3390/polym15061552)
Supplement: Supplementary file 1 [file polymers-15-01552-s001.zip › polymers-2137394-supplementary.pdf]

polymers

MDPI

Supplementary Materials

S1. Materials

The materials used in this study are summarized in Table S1. The polymer matrix was thermoplastic polyurethane (TPU) for injection moulding and powder bed fusion (3D printing), and epoxy for casting. Three different types of hexagonal BN (hBN) powder were used for preparation of hBN/polymer composites.

Results presented in the article are based on the polymer composites containing BN3. Sections 9 and 10 of this Supplementary Materials provide additional results of the polymer composites containing BN1 and BN2. This supports the discussion and evaluation of the effects of filler (e.g. particle size, as well as single platelets vs. partly agglomerated particles) on the thermal conductivity and tensile properties of the hBN/polymer composites.

Table S1. Materials used for preparation of hBN/polymer composites.

Name	Materials	Product name, Supplier
	hBN particles	
BN1	hBN powder with average particle size of ~ 1 µm	Boron nitride powder, 1 µm, 98% (product no. 255475),<br>Sigma Aldrich, Merck
BN2[a]	hBN powder. Single platelets with median particle size (D_{50}) of 12 µm and size distribution in the range (0.8–40) µm, specific surface area (BET) ~ 6 m^2/g	HeBoFill LL-SP 120,<br>Henze Boron Nitride Products AG, Germany
BN3[a]	hBN powder. Platelet agglomerates with D_{50} of 20 µm and size distribution in the range (0.5–31) µm, BET ~ 4 m^2/g	HeBoFill CL-ADH 020,<br>Henze Boron Nitride Products AG, Germany
	Polymer matrix	
TPU	Ultrasint TPU 88A (in the form of powder)	BASF, Germany
Epoxy[b]	An epoxy system (for casting) containing:<br>- 35 wt% unmodified bisphenol-F epoxy resin (Araldite GY 285-1)<br>- 35 wt% reactive diluent (Araldite DY 026)<br>30 wt% amine-based curing agent (Jeffamine D-230 Polyetheramine)	Huntsman

[a]Information about BN2 and BN3 as provided by the supplier [1,2]. Size distributions of BN2 and BN3 are shown in Figure S1. The powder BN3 had a partly agglomerated particle structure, claimed to give good lubricating properties and low viscosity increase [2]. Platelets and spherical agglomerates are shown in Figure S2a.[b]The epoxy system was formulated to have a low viscosity, suitable for preparing composites with high filler content.

S2. Specimen Fabrication

All specimens, with codes, are listed in Table S2 and Table S3. Details about the fabrication by casting and powder bed fusion (PBF) are given below.

Table S2. Specimens fabricated by injection moulding and casting.

Specimen ID	Processing technique	Polymer	BN type	hBN content (wt%)
IM_TPU	Injection moulding	TPU	BN3	0
IM_15BN3				15
IM_25BN3				25
IM_35BN3				35
IM_50BN3				50
IM_65BN3				65
IM_15BN2			BN2	15
IM_35BN2				35
IM_50BN2				50

IM_50BN1			BN1	50
C_Epoxy				0
C_35BN3				35
C_50BN3	Casting	Epoxy	BN3	50
C_55BN3				55

Casting:

Composite specimens of epoxy and hBN were prepared by vacuum mixing (Thinky Planetary Vacuum Mixer) followed by casting into a PTFE mould and curing at 150 ºC for 18 hours. The epoxy system contained 35 wt% bisphenol-F epoxy, 35 wt% diluent and 30 wt% curing agent (details in the article). This formula was selected to achieve a low viscosity, suitable for preparing composites with a high filler concentration. The epoxy system was mixed with hBN powder in vacuum through four steps, in order to avoid air trapped inside the mixture during mixing. The time for each mixing step was 2 minutes; the rotation speed was 2000 rpm for the first two steps and 1500 rpm for the last two steps; and vacuum was decreased from 50 kPa to 30 kPa, 10 kPa and 2.5 kPa from the first step to the fourth step. The maximum practical filler content was about 55 wt%, for which there was no formation of voids or air bubbles during mixing and casting.

Powder Bed Fusion (PBF):

A desktop PBF 3D printer (SnowWhite, Sharebot, Italy) was employed to fabricate specimens from TPU powder and hBN/TPU powder mixtures. The machine has a maximum build volume of 100 mm x 100 mm x 100 mm, a CO_2 laser (wavelength 10.6 μm, max. power 14 W), a blade for recoating powder (i.e. applying a new layer of powder). The printing was performed in air.

In order to identify appropriate processing parameters for the hBN/TPU composites, the starting point was the parameters suggested for the TPU by the material supplier (for other machines than the one used in this study). However, a higher laser energy density was needed, also for the pure TPU. In addition, a higher hBN content required a higher energy, see Table S3. However, composites with satisfactory fusion (i.e. with enough ductility for practical use) could not be obtained by PBF, at least not with the present powder mixing method and recoater.

Table S3. Specimens fabricated by PBF 3D printing. The process parameters E_V, P, v and L are explained in the article. The laser energy (E_V) was applied three times for each powder layer, i.e. three repeated laser scans (the recommendation from the material supplier was to apply two laser scans). The hatching distance (the distance between two adjacent laser scanning lines for the contour lines and the infill lines) is not included in the table. It was kept constant at 0.1 mm.

**Specimen ID**	**BN content (wt%)**	***E_V* (J/mm^3)**	***P* (W)**	***v* (m/s)**	***L* (mm)**	**Chamber temp. (ºC)**	**Specimen orientation**
PBF_TPU	0	0.31	8.4	2.7	0.10	88	xy
			**BN type 2**				
PBF_15BN2	15	0.13	3.5				
PBF_25BN2	25	0.21	5.6	2.7	0.10	88	xy
PBF_35BN2	35	0.31	8.4				
			**BN type 1**				
PBF_35BN1	35	0.31	8.4	2.7	0.10	88	xy
			**BN type 3**				
PBF_35BN3	35	0.31	8.4				
PBF_40BN3_a		0.26	7.0				
PBF_40BN3_b		0.31		2.7			xy
PBF_40BN3_b_z		0.31				88	z
PBF_40BN3_b_92		0.31				92	
PBF_40BN3_c		0.35	8.4	2.4	0.10		

PBF_40BN3_d_08	40	0.39			0.08		
PBF_40BN3_d		0.39		2.7			xy
PBF_40BN3_d_z		0.39	10.5				z
PBF_40BN3_e		0.50		2.1	0.10	88	xy

S3. hBN Powders and hBN/polymer Composites Characterized by SEM

Two types of hBN powders from Henze (BN2 and BN3) were used. Particle size distributions of BN2 (single platelets) and BN3 (partly agglomerated) are shown in Figure S1. Platelets and spherical agglomerates of BN3 can be seen in Figure S2a. Figure S2a shows a cast specimen with 55 wt% BN3. A PBF composite printed in the xy plane (specimen PBF_40BN3_d) is shown in Figure S2b, revealing voids.

These SEM micrographs were obtained using Secondary Electron (SE) detectors. In cases where low vacuum was used to minimize image distortion from sample charging, SE were recorded by an “Ultra Variable-pressure Detector” (UVD), which indirectly detects SE emission. Cross-sections were cut along the (2 mm) thickness direction of injection moulded and PBF discs. Similar cross-sections of cast discs were prepared by etching, using an Ar ion milling system (Hitachi IM 4000). The ion etching was conducted with 6 kV accelerating voltage, in cross-section mode (a Ta mask was used for defining the cross-section).

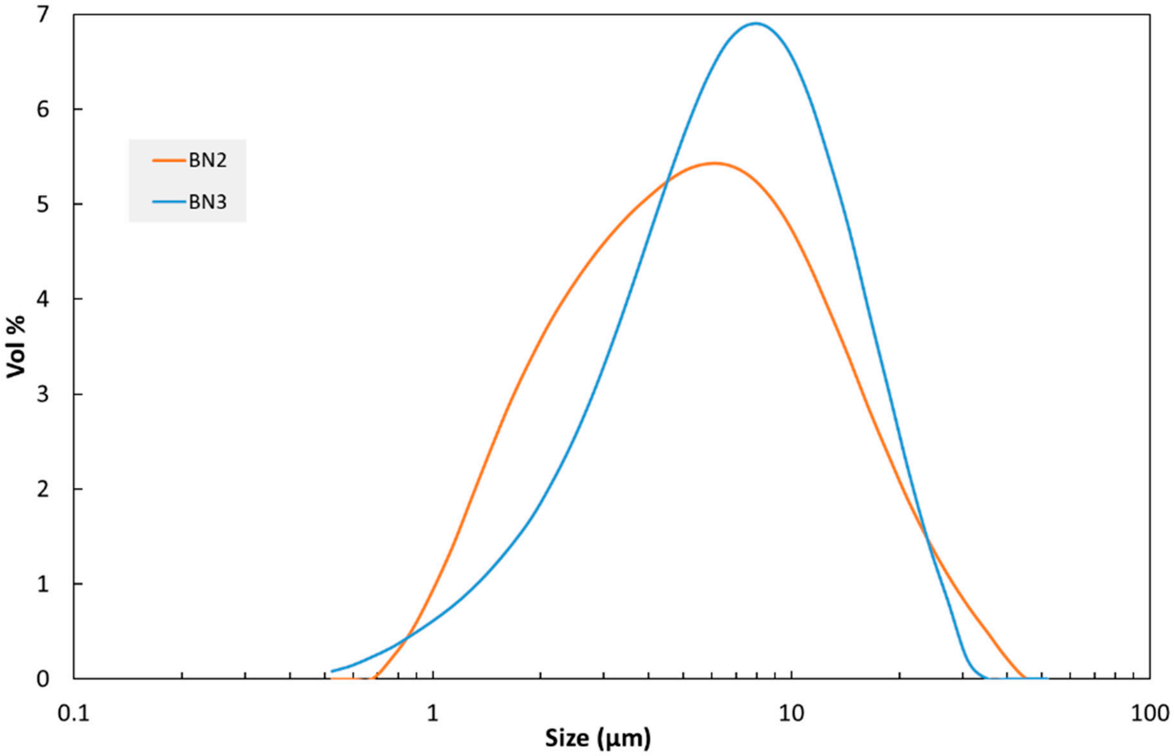

Figure S1. Particle size distribution of BN2 and BN3. Data from powder supplier.

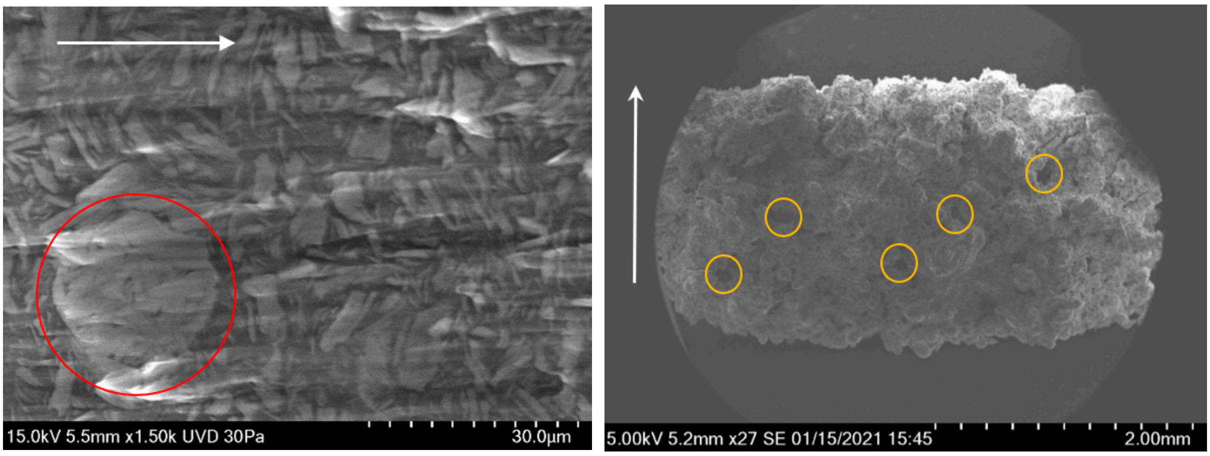

(a) (b)

Figure S2. SEM micrographs of cross-sections of disc specimens. The white arrows show the direction of the disc normal. The bright spots are artefacts caused by charging in the SEM. (a) Ion milled cross-section of disc C_55BN3**.** An agglomerate of hBN platelets is seen inside the red circle. (b) Cross-section of PBF disc printed in the xy plane (specimen PBF_40BN3_d). The yellow circles show the positions of voids.

S4. Determination of Density for the Thermal Conductivity Measurements

The density of some PBF specimens (e.g. PBF_TPU; PBF_15BN2; PBF_25BN2; PBF_35BN2; PBF_35BN3; PBF_40BN3_c; PBF_40BN3_c_z) was measured gravimetrically (Mettler Toledo XS204 with density kit). The measurements were conducted at 20 ºC, using water as the immersion liquid. All specimens were measured twice, and they were dried well between each time. The difference between the measured density and the calculated density (based on mass and dimensions) was negligible. Examples: the measured and calculated densities of specimen PBF_40BN3_c were 0.73 g/cm^3 and 0.75 g/cm^3, respectively. For PBF_TPU, the measured and calculated densities were 0.85 g/cm^3 and 0.84 g/cm^3. Therefore, calculated densities were used to determine thermal conductivity of all (remaining) samples.

S5. Tensile Testing – Supplementary Experimental Details and Examples of Stress-Strain Curves

Tensile testing of injection moulded specimens was performed with a universal test machine (Zwick Z250), using a 2.5 kN load cell. The specimen was mounted in wedge grips, with grip-to-grip distance of 55 mm. Strains were calculated from displacements measured with an extensometer with initial gauge length of 25 mm. The crosshead speed was 0.5 mm/min up to a strain of 0.25 %, and then changed to 25 mm/min (as suggested described in ISO 527-1:2012). These speeds were chosen in order to have nominal strain rates similar to those typically used in tensile testing of plastics, with the most common (larger) test specimen type 1A of ISO 527-2. PBF specimens were tested with a smaller machine (Lloyd Instruments LR50K) using a 500 N load cell. The crosshead speed was constant (25 mm/min) and an extensometer was not used. Three specimens were tested for each filler concentration, and the reported data are average values.

Figure S3 shows an example of tensile stress-strain curves. These curves illustrate that the two composites with 50% BN2 and BN3, respectively, differ with regard to strength (maximum stress) and strain at break. The differences are small, but statistically significant. See also Figures S9 and S10 below.

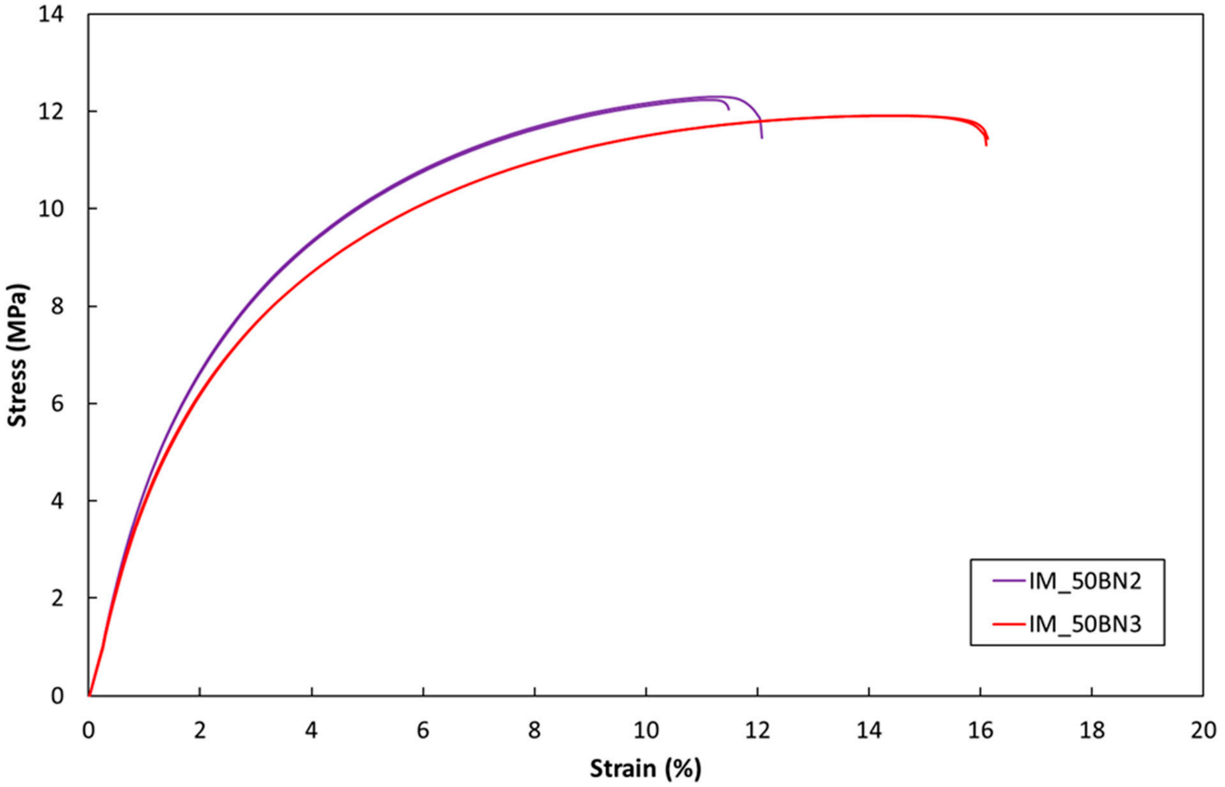

Figure S3. Tensile stress-strain curves for the composites IM_50BN2 and IM_50BN3. Curves for two repeated tests are shown for each composite.

S6. Specimen preparation for XRD of the specimen's mid-plane

Injection-moulded disc specimens were cast in epoxy. To expose the mid-plane for XRD measurement, the specimens were grinded to their half-thickness (1 mm) and polished. XRD data obtained with the half-thickness specimen (referred to as IM_BN3_center), with the X-ray beam towards the mid-plane mentioned above, was compared with data obtained with the original 2 mm thick specimen, in order to evaluate the orientation of hBN platelets in the 'skin' and core regions of injection moulded specimens.

S7. Numerical simulation of platelet orientation in injection moulded discs

The process-induced orientation of plate-like particles in the ø25 mm 2 mm thick disc was simulated with the commercial software Moldex3D (release R2020). The simulation of the injection moulding process involves non-isothermal flow of a non-Newtonian fluid. The platelet orientation was simulated with the improved anisotropic rotary diffusion and retarding principal rate (iARD-RPR) model [3]. This model has four parameters: the effective Jeffery aspect ratio of a cylindrical particle (e.g. plate) (R), a particle–particle interaction parameter (C_I), a particle-matrix interaction parameter (C_M) and a retardation parameter (α). Identifying and verifying model parameters for predicting the orientation of hBN platelets is beyond the scope of this paper, but a small study was performed to assess the sensitivity of the simulated orientation to the four parameters mentioned above, and to the viscosity of the polymer and some moulding parameters.

Numerical simulations were performed to obtain the 3D distributions platelet orientation in the injection moulded disc. As we do not have a verified material model for the orientation of these hBN platelets in a flowing polymer melt (i.e. verified parameters for the iARD-RPR model), the purpose of the simulations is mainly to obtain a qualitative picture of how the orientation varies through the thickness of the disc, in order to compare with the $\langle cos^2\theta \rangle$ values from XRD, and $\langle cos^2\theta \rangle$ values used in two of the models for thermal conductivity (section 4.4 in the article).

Figure S4 shows how the orientation varies through the thickness of the disc, at the center point of the disc. The $\langle cos^2\theta \rangle$ value (blue curve, Az) is highest in a "shell" near the surface, and lowest in the midplane/core of the disc. In a skin layer (about 50 µm thick in Figure S4) the orientation decreases towards the surface. This can be attributed to the fountain flow at the flow front and/or thermal effects [4]. The red curve (Ax) in Figure S4 is the orientation of the platelet normal relative to the x axis, i.e. the main flow direction, see Figure S5. Near the surface this value is lower than 0.1, i.e. the platelet normals are essentially perpendicular to the x direction. In the core, the value is 0.4, i.e. the platelet normals have a slightly preferred orientation along the x axis (a random orientation would give Ax = 1/3).

Figure S5 shows how the orientation ($\langle cos^2\theta \rangle$) varies through the thickness of the disc, in different "slices" through the xy plane. The thickness of the shell and core layers, as well as the maximum and minimum values, varies over the disc area, especially along the main flow path from the gate to the other side of the disc (slice A in Figure S5).

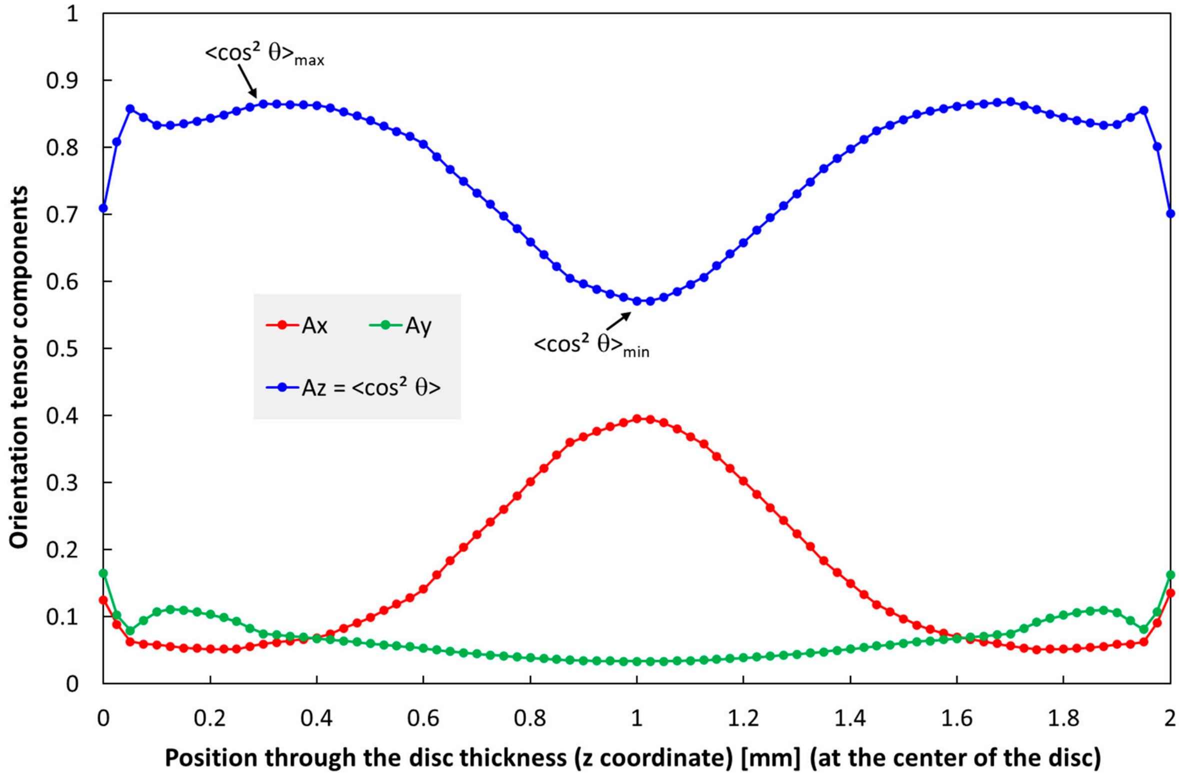

Figure S4. Simulated platelet orientation through the thickness of the 2 mm thick disc; diagonal components of the second order orientation tensor. The three components/curves relate to the coordinate system in Figure S5. The component Az corresponds to $\langle cos^2\theta \rangle$ in other sections of the paper. Parameters for the iARD-RPR orientation model: R = 0.05, C_I = 0.005, C_M = 0, α = 0.7. Note that this model does not have a parameter for the platelet fraction as such.

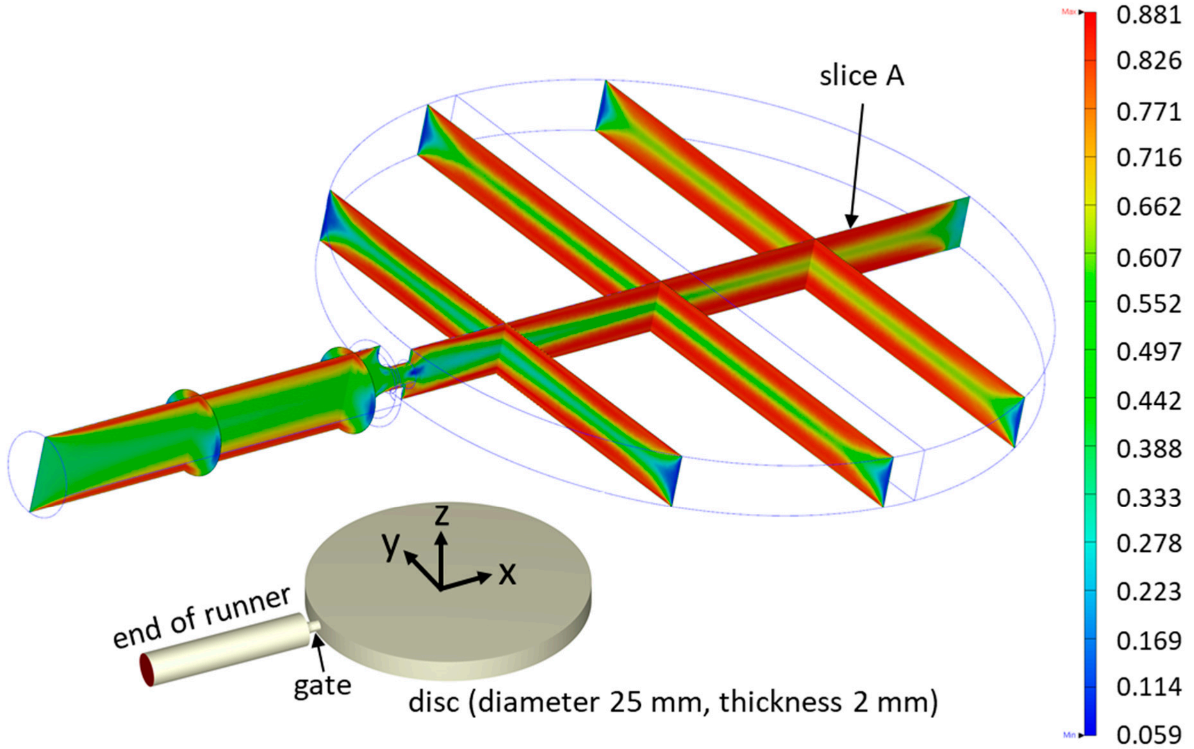

Figure S5. Simulated platelet orientation (Az = $\langle cos^2\theta \rangle$) in the 2 mm thick disc. The orientation data are shown in slices through the thickness of the disc, including gate and part of the runner. The model parameters are the same as in Figure S4.

The sensitivity of the simulated orientation to model parameters was also assessed. The main parameter affecting the maximum, minimum and average $\langle cos^2\theta \rangle$ through the thickness of the disc is the particle–particle interaction parameter (C_I). Typically, the $\langle cos^2\theta \rangle_{max}$ values are in the range 0.85-0.97 and the average values are in the range 0.75-0.85.

Here we will only summarize a few trends, based on the orientation through the thickness at the center of the disc, as in Figure S4 .

The main parameter affecting $\langle cos^2\theta \rangle_{max}$ is the particle–particle interaction parameter (C_I); $\langle cos^2\theta \rangle_{max}$ increases with decreasing C_I. It also increases with decreasing platelet aspect ratio (R) (however, R must differ from the experimental value to have a significant effect) and increasing matrix-particle interaction parameter (C_M). Typically, the $\langle cos^2\theta \rangle_{max}$ values are in the range 0.85-0.97.

The $\langle cos^2\theta \rangle_{min}$ value is also affected by the parameters, but less than the maximum value. Just as the maximum value, $\langle cos^2\theta \rangle_{min}$ also increases with decreasing C_I, but it is insensitive to R. The difference between the maximum and minimum increases with decreasing C_I, and C_I is the dominant parameter.

The average of $\langle cos^2\theta \rangle$ through the thickness increases with decreasing C_I, and C_I is the dominant parameter. This average is typically in the range 0.75-0.85.

The viscosity of the polymer also influences the orientation. The main effects of increasing the viscosity (via the zero-shear viscosity in the viscosity model) are to reduce the minimum and average $\langle cos^2\theta \rangle$ through the thickness. However, for a typical viscosity range, the average $\langle cos^2\theta \rangle$ is only about 0.04 higher or lower than the average values given above.

The injection moulding process parameters also affect the orientation. In the simulations referred to above, the fill time for the disc was set to 0.2 s, which is close to the experimental value. (Other moulding parameters in the simulations were set as in the experiment.) If the fill time is increased to 1.2 s in the simulation, $\langle cos^2\theta \rangle_{max}$ decreases, while $\langle cos^2\theta \rangle_{min}$ increases. The net effect is that the average $\langle cos^2\theta \rangle$ through the thickness

increases slightly – e.g. from 0.77 to 0.81 with the model parameters listed in the caption of Figure S4 .

S8. Differential Scanning Calorimetry (DSC) Results

DSC of the pure TPU and TPU with 40 wt% BN3 was performed using a Perkin Elmer DSC 8500. The heating/cooling rate was 10 K/min, and the samples were heated twice from 25 °C to 255 °C in nitrogen atmosphere. Thermograms of TPU powder and TPU powder with 40 wt% BN3 are shown in Figure S6. The DSC thermograms showed that the TPU with 40 wt% BN3 had a broad melting endotherm, from about 120 ºC to 154 ºC, with a peak at about 133 ºC. The crystallization had a broad exotherm from about 120 ºC to 98 ºC, with a peak at about 109 ºC. The difference between the composite and pure TPU was small (about 2 ºC). Therefore, the temperatures in the PBF process can be the same for pure TPU and hBN/TPU composites.

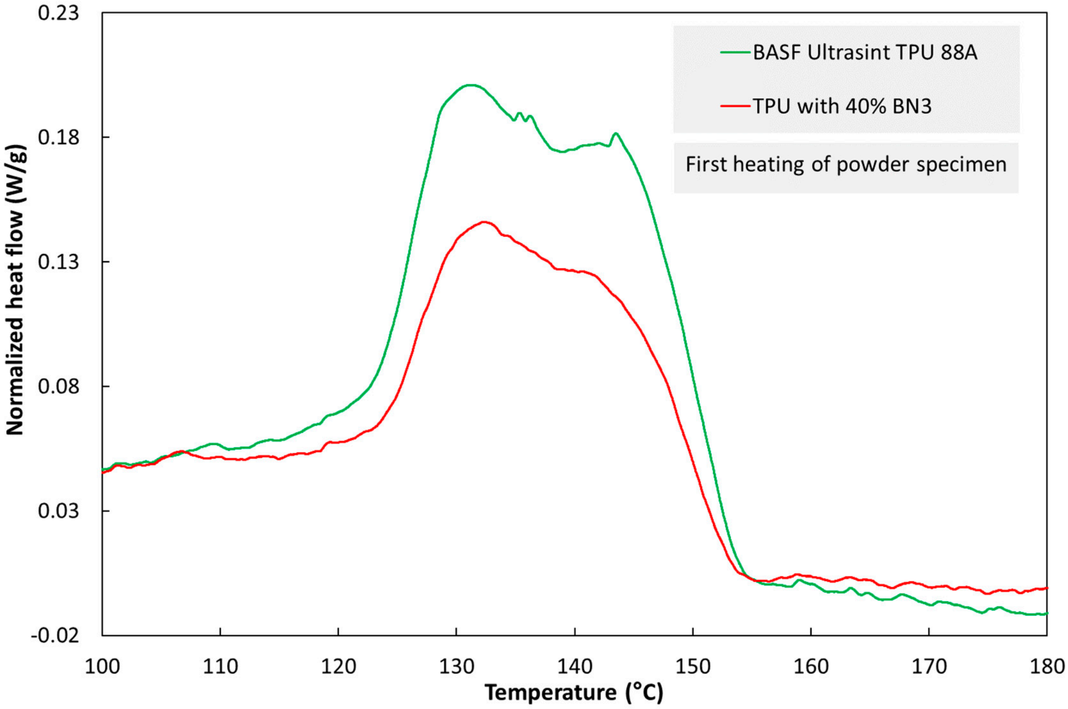

(a)

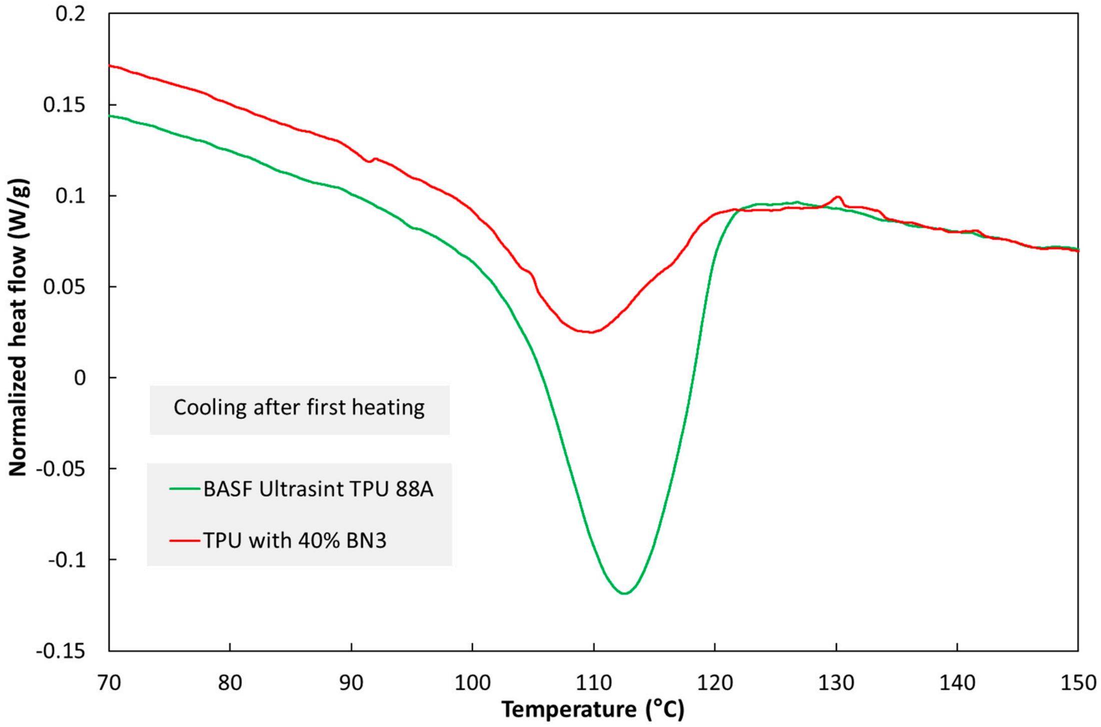

(b)

Figure S6. DSC thermograms of the pure TPU power and the TPU powder with 40 wt% BN3 from (a) heating scans and (b) cooling scans.

S9. Additional Results for Thermal Conductivity with Two other hBN Types (BN1 and BN2)

In addition to BN3 (used in the article), two other filler types (BN1 and BN2, see Supplementary section S1) were used to study the thermal conductivity of hBN/TPU composites. The additional results in this section supplements and supports the discussion in the article, regarding the effects of filler (e.g. particle size, as well as single platelets vs. partly agglomerated particles) on the thermal conductivity of the hBN/polymer composites.

Figure S7 shows that for IM composites with 50 wt% hBN, there was a clear effect of hBN type; BN2 and BN1 gave the highest and lowest conductivity, respectively. The same trend was seen for the PBF composites, although the effect was weaker.

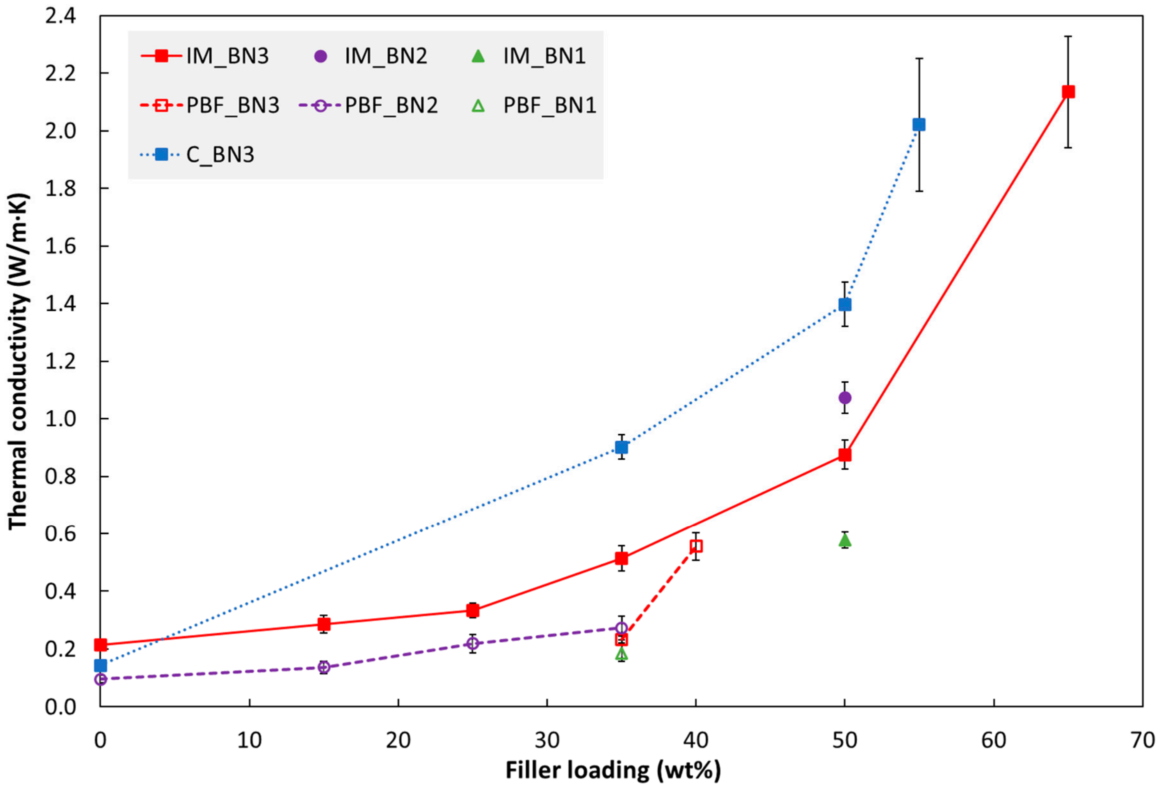

Figure S7. Thermal conductivity (at 30 ºC) of composites fabricated by injection moulding ("IM"), powder bed fusion ("PBF") and casting ("C") as a function of hBN loading. The PBF specimen with 40 wt% BN3 was processed with a higher laser energy density than the other PBF specimens in this figure, see Figure 3 in the article and Table S3.

S10. Additional Results for Tensile Properties with Another hBN type (BN2)

In addition to hBN type BN3 (used in the article), another type (BN2, see Supplementary section S1) was also used in tensile tests. The additional results supports and supplements the discussion (in the article) regarding effects of filler (e.g. particle size, as well as single platelets vs. partly agglomerated particles) on the material processing and tensile properties.

The tensile properties of injection moulded specimens containing hBN type BN2 and BN3 are shown in Figure S8 – Figure S10. The tensile modulus increases monotonously with increasing filler loading in this range. The strength and strain at break values are almost unaffected by adding 15 % hBN. With 35 % hBN, both these values are reduced. With 50% hBN, the strength values are higher than that for 100 % TPU, while the strain at break values are similar to those for 35 % hBN. With 65 % hBN (only BN3) the highest strength and the lowest strain at break are observed. There are small, but statistically significant, differences between composites with BN2 and BN3, see also Figure S3.

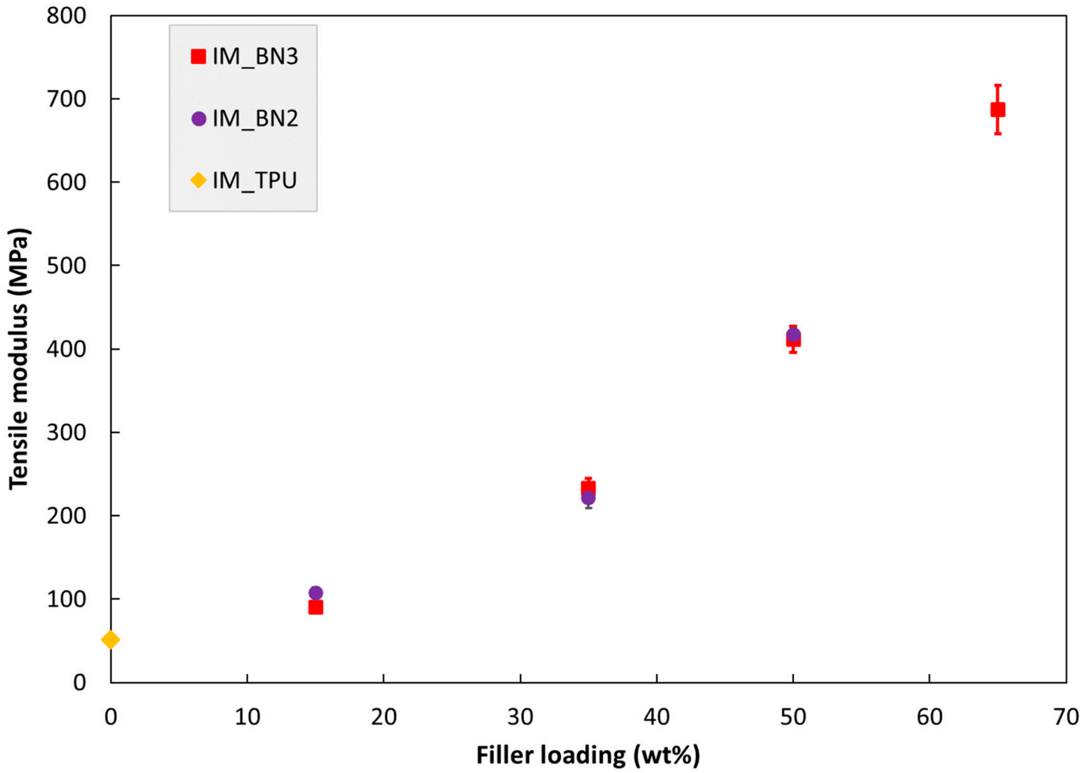

Figure S8. Tensile modulus of injection moulded specimens as a function of hBN loading. The specimen IM_TPU is 100% TPU.

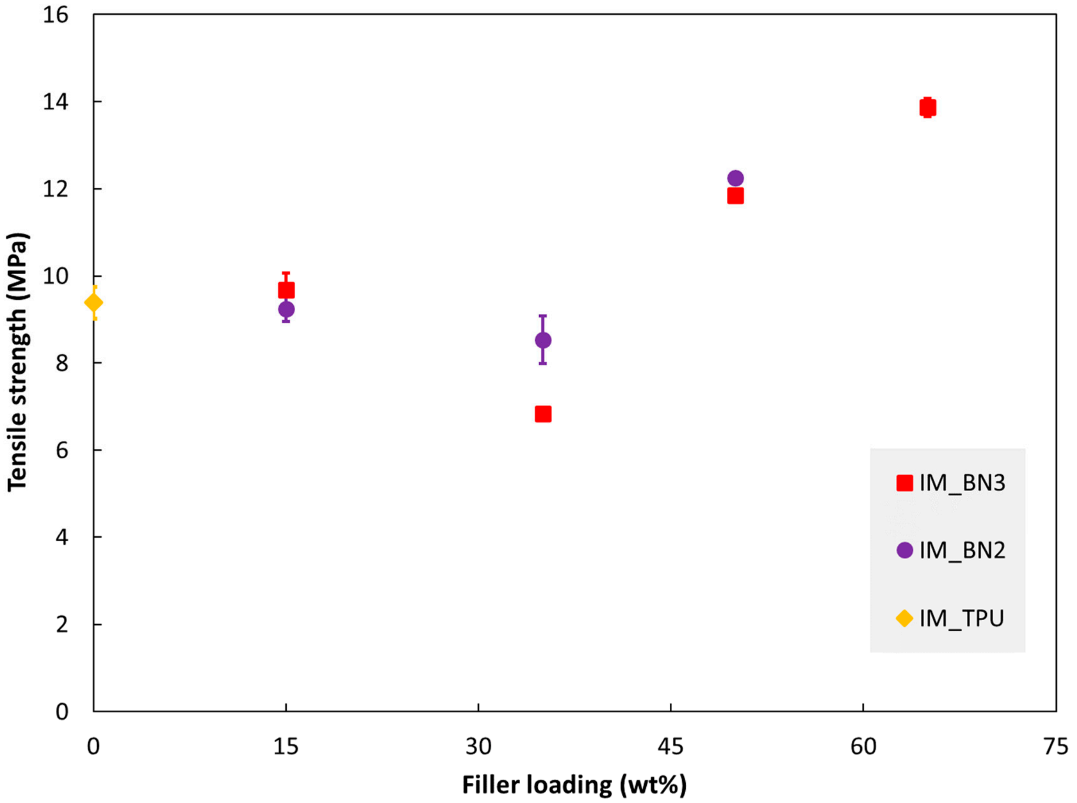

Figure S9. Tensile strength of injection moulded specimens as function of hBN loading.

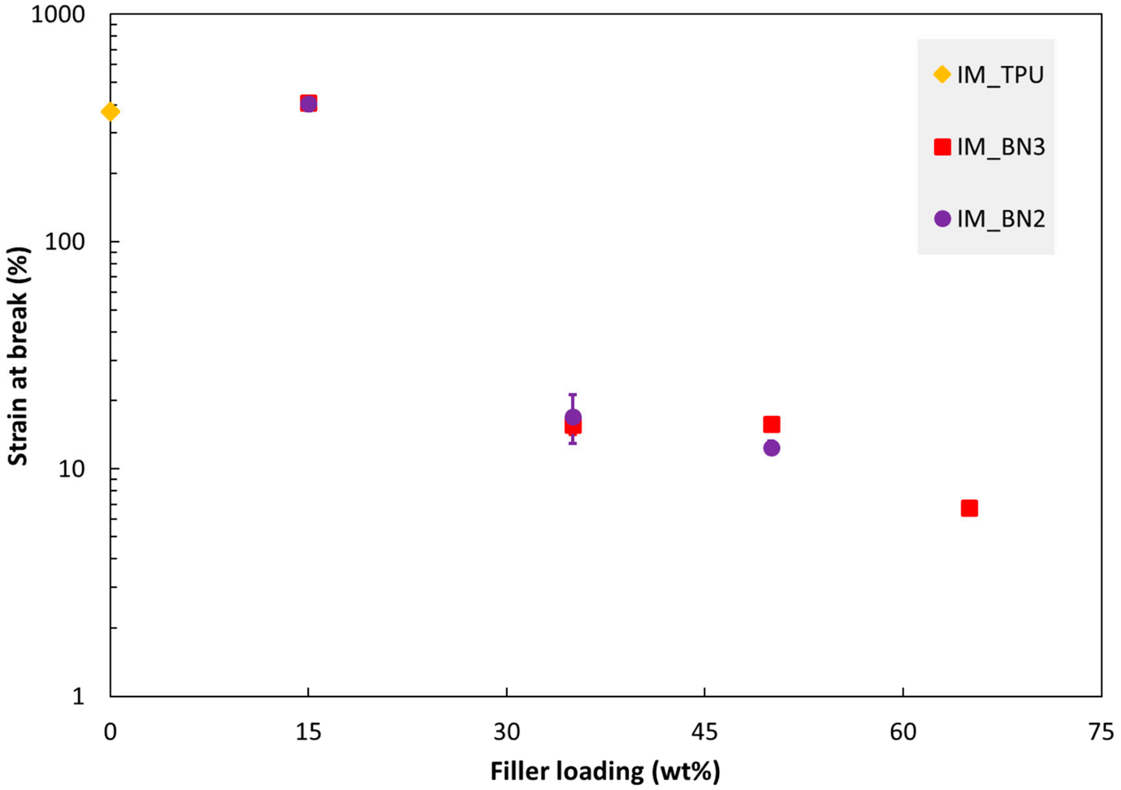

Figure S10. Strain at break of injection moulded specimens as a function of hBN loading.

S11. The Model of Nan et al.

The experimental thermal conductivities in this article were analyzed by comparing with the effective medium approximation (EMA) model introduced by Nan et al. [5]–[8]. This model, for the thermal conductivity of composites, includes effects of particle shape (ellipsoids, which can represent platelets), particle orientation and interfacial thermal resistance. It has been used in some studies of composites with hBN. However, it is limited to particles with isotropic thermal conductivity, while our hBN platelets are anisotropic. Also note that the model does not take into account filler-filler contact or nonuniform particle distributions. Hence, it is restricted to low filler fractions, and it can not predict the percolation threshold.

This supplementary section provides details about this model and background for the parameters used in our study.

In the model of Nan et al. [5], the through-plane thermal conductivity (K_{33}) of a composite with oblate ellipsoidal particles ($p < 1$) (which can represent platelets) is expressed by Eq. S1:

$$K_{33} = K_m \frac{1 + f[\beta_{11}(1 - L_{11})(1 - \langle cos^2\theta \rangle) + \beta_{33}(1 - L_{33})\langle cos^2\theta \rangle]}{1 - f[\beta_{11}L_{11}(1 - \langle cos^2\theta \rangle) + \beta_{33}L_{33}\langle cos^2\theta \rangle]} \quad \text{(S1a)}$$

with:

$$L_{11} = L_{22} = \frac{p^2}{2(p^2 - 1)} + \frac{p}{2(1 - p^2)^{3/2}} cos^{-1}p \quad \text{(S1b)}$$

$$L_{33} = 1 - 2L_{11} \quad \text{(S1c)}$$

$$p = \frac{a_3}{a_1} \quad \text{(S1d)}$$

$$\beta_{ii} = \frac{K_{ii}^C - K_m}{K_m + L_{ii}(K_{ii}^C - K_m)} \quad \text{(S1e)}$$

$$K_{ii}^C = \frac{K_p}{1 + \frac{\gamma L_{ii} K_p}{K_m}} \quad \text{(S1f)}$$

$$\gamma = (1 + 2p)\frac{R_{BD} K_m}{a_3} \quad \text{(S1g)}$$

$$\langle cos^2\theta \rangle = \frac{\int \rho(\theta) cos^2\theta sin\theta d\theta}{\int \rho(\theta) sin\theta d\theta} \quad \text{(S1h)}$$

where:

K_m and K_p are the **thermal conductivities** of the matrix and particles (hBN platelet in our case), respectively. The K_m value is taken from the measured data; 0.22 W/m·K for the injection moulded TPU and 0.14 W/m·K for the cast epoxy. The K_p value used is the average thermal conductivity of hBN (300 W/m·K), see also sensitivity analyses below. The hBN platelet has anisotropic thermal conductivity due to its layered structure [8]. The in-plane thermal conductivity (along the (002) plane) of hBN is about 600 W/m·K, due to strong covalent bonds between B atoms and N atoms. The through-plane thermal conductivity (along the (100) plane) is around 2–30 W/m·K [9]. The K_p value has a minor effect on the thermal conductivity of the injection moulded composites, which have $\langle cos^2\theta \rangle$ in the range 0.75 to 1, as shown in Figure S12.

f is the volume fraction of particles (vol%)

K_{ii}^c is the **equivalent thermal conductivity** along the *ii* symmetric axis of the composite unit cell (with 11 and 33 representing in-plane and through-plane directions, respectively)

L_{ii} are **geometrical factors**, dependent on the particle shape (on our case: $L_{11} = L_{22} = 0.037$; $L_{33} = 0.926$)

p is the **ellipsoid particle aspect ratio**; in our case a_1 is the platelet diameter and a_3 is the thickness. According to information from the hBN powder supplier, a_1 and a_3 of powder BN3 are about 20 μm and 1 μm, respectively. Hence, the value of p used for the model is 0.05.

R_{BD} is the **interfacial thermal resistance** between particle and matrix (a property concentrated on a surface with zero thickness). The R_{BD} range of (10^{-8} – 10^{-6}) m^2W/K for the hBN/polymer composites have been found by previous studies on polymer composites with hBN [6]–[8], [10]. Hence, the R_{BD} values from 0 to 10^{-6} m^2W/K are used in our paper.

$\langle cos^2\theta \rangle$ is a measure of the **orientation of the ellipsoid particles** in the matrix (platelets in our case); θ is the angle between a platelet surface normal and the specimen surface normal; $\rho(\theta)$ is a distribution function describing the platelet orientation. Hence, $\langle cos^2\theta \rangle$ is 1, 1/3 or 0 for platelets with in-plane, random or through-plane orientation, respectively (as illustrated in Figure S11) [5]. The $\langle cos^2\theta \rangle$ values in our study are based on the data from the XRD measurements of the IM and cast specimens, as presented in Figure 11 in the article. The cast (hBN/epoxy) specimens have $\langle cos^2\theta \rangle$ values in the range (0.4 – 0.65) for hBN loadings in the range (21.6 – 38.5) vol%. For the IM (hBN/TPU) specimens, the $\langle cos^2\theta \rangle$ values of composites with (8.3 – 14.6) vol% hBN are about 0.85, and composites with (21.6 – 48.7) vol% hBN have average $\langle cos^2\theta \rangle$ values in the range of (0.75 – 0.85).

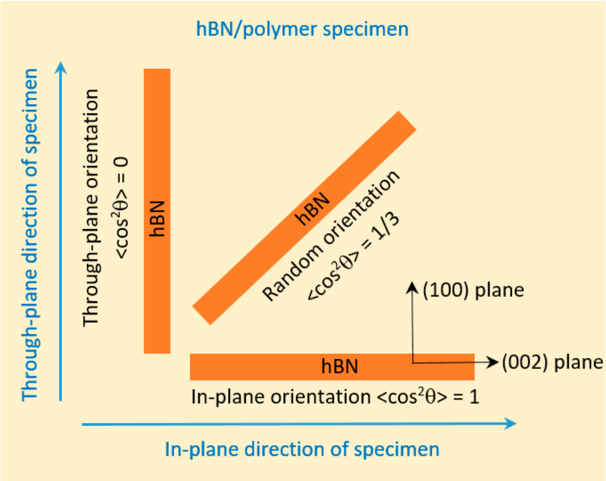

Figure S11. Orientation of hBN platelets; through-plane, random and in-plane.

There are uncertainties in our data with regard to using this model to interpret our experimental results. For instance, the interfacial thermal resistance is unknown, and the average orientation of platelets through the cross section is uncertain, at least for injection moulded specimens. Other parameters are also uncertain, such as the size and aspect ratio of the platelets.

In order to evaluate these limitations and uncertainties, the effect of various parameters on the model through-plane conductivity (K_{33}) are shown in Figure S12a-c. In each diagram (a to c), K_{33} is plotted as function of platelet loading for selected values of platelet conductivity (K_p = 30, 300 and 600 W/m·K) and average platelet orientation ($\langle cos^2\theta \rangle$ = 1/3, 0.75 and 1). The interfacial thermal resistance (R_{BD}) is 0, $3{\cdot}10^{-7}$ and 10^{-6} m^2W/K, in Figure S12a, Figure S12b and Figure S12c, respectively.

Figure S12 shows that for $\langle cos^2\theta \rangle$ = 1 (in-plane orientation of platelets), the K_{33} value is the same for the three selected K_p values. The effect of R_{BD} is also small.

For $\langle cos^2\theta \rangle$ = 0.75, the K_{33} value is practically the same whether K_p is set to 300 or 600 W/m·K, while K_{33} is reduced when K_p is reduced to 30 W/m·K (although the reduction in K_{33} is small for platelet fractions below about 20 vol%). The effect of R_{BD} is noticeable for this orientation. For K_p set to 300 or 600 W/m·K, K_{33} (at 48.7 vol% hBN) is reduced from about 1.75 to about 1.25 W/m·K when R_{BD} is increased from 0 to 10^{-6} m^2W/K.

Also for the lowest orientation in Figure S12, i.e. $\langle cos^2\theta \rangle$ = 1/3, K_{33} is nearly independent of K_p in the range 300 or 600 W/m·K, but reducing K_p to 30 W/m·K has a quite large effect on K_{33}. The sensitivity to R_{BD} is also higher for this orientation.

Hence, for our injection moulded specimens, with $\langle cos^2\theta \rangle$ in the range 0.75 to 1, a K_p value of 300 W/m·K can be used, although K_{33} is slightly overpredicted with this K_p value. However, it is difficult to estimate the R_{BD} value via model fits, due to uncertain average $\langle cos^2\theta \rangle$ values through the specimen thickness and uncertain platelet dimensions, as well as potential platelet-platelet contact or nonuniform platelet distributions or voids. Anyhow, comparing experimental data with model predictions with different R_{BD} and $\langle cos^2\theta \rangle$ values can give some insight.

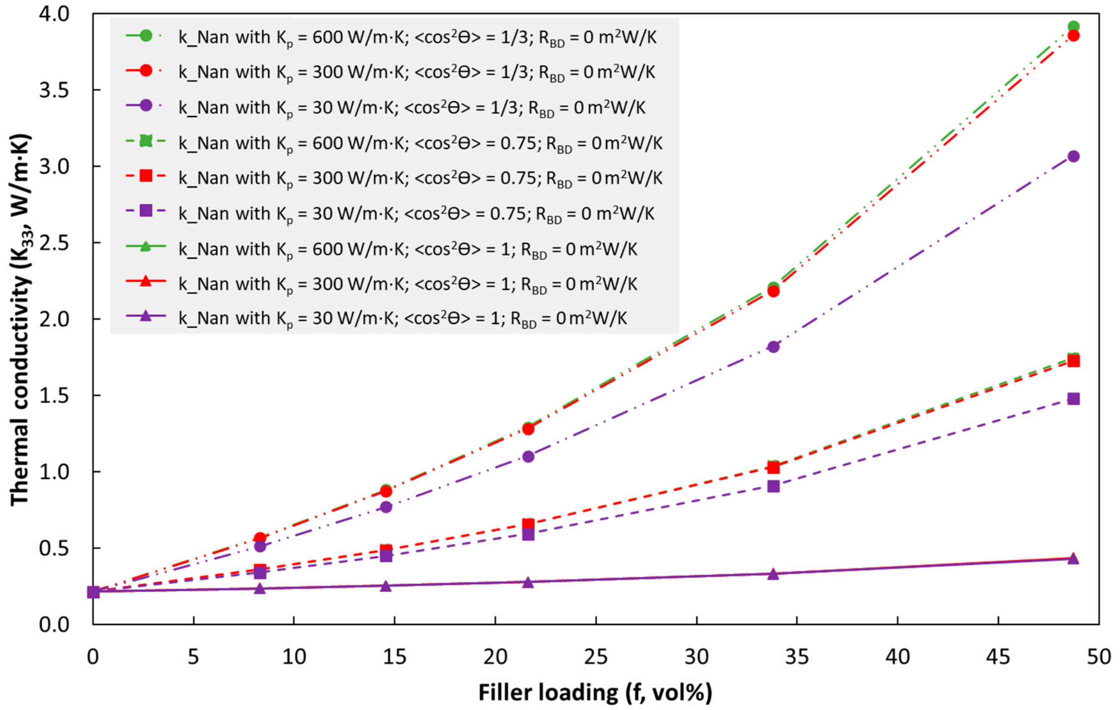

4.0
3.5
3.0
2.5
2.0
1.5
1.0
0.5
0.0
0
5
10
15
20
25
30
35
40
45
50
Thermal conductivity (K33, W/m·K)
Filler loading (f, vol%)
k_Nan with Kp = 600 W/m·K; <cos2Θ> = 1/3; RBD = 0 m2W/K
k_Nan with Kp = 300 W/m·K; <cos2Θ> = 1/3; RBD = 0 m2W/K
k_Nan with Kp = 30 W/m·K; <cos2Θ> = 1/3; RBD = 0 m2W/K
k_Nan with Kp = 600 W/m·K; <cos2Θ> = 0.75; RBD = 0 m2W/K
k_Nan with Kp = 300 W/m·K; <cos2Θ> = 0.75; RBD = 0 m2W/K
k_Nan with Kp = 30 W/m·K; <cos2Θ> = 0.75; RBD = 0 m2W/K
k_Nan with Kp = 600 W/m·K; <cos2Θ> = 1; RBD = 0 m2W/K
k_Nan with Kp = 300 W/m·K; <cos2Θ> = 1; RBD = 0 m2W/K
k_Nan with Kp = 30 W/m·K; <cos2Θ> = 1; RBD = 0 m2W/K

(a).

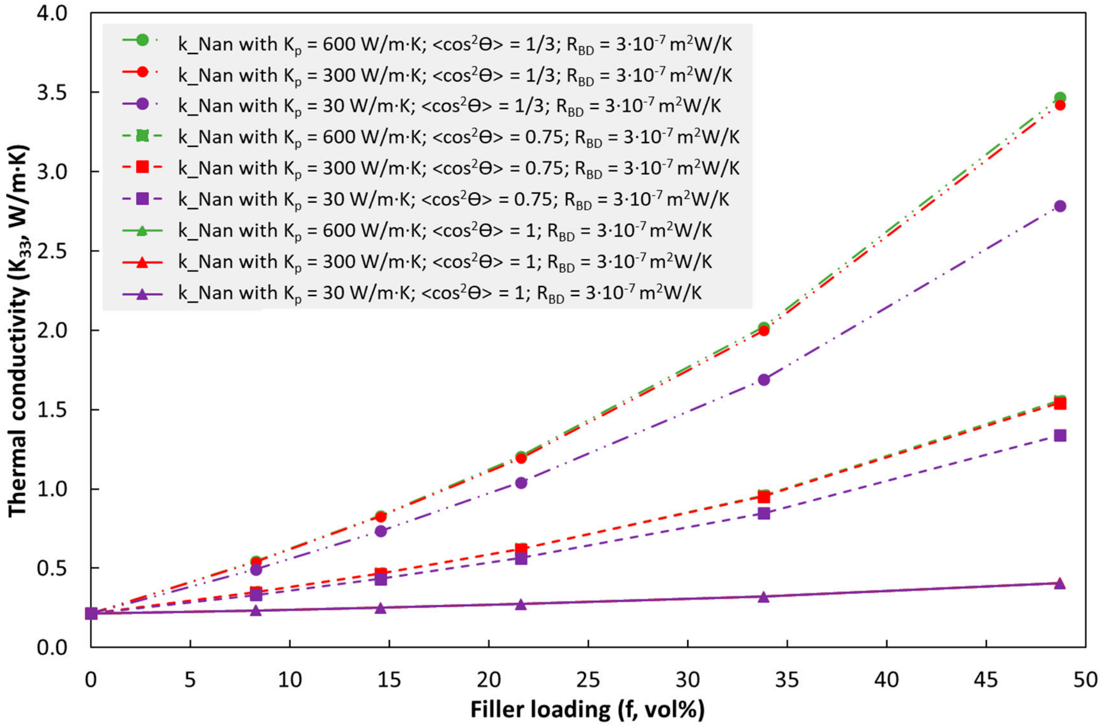

4.0
3.5
3.0
2.5
2.0
1.5
1.0
0.5
0.0
0
5
10
15
20
25
30
35
40
45
50
Thermal conductivity (K33, W/m·K)
Filler loading (f, vol%)
k_Nan with Kp = 600 W/m·K; <cos2Θ> = 1/3; RBD = 3·10-7 m2W/K
k_Nan with Kp = 300 W/m·K; <cos2Θ> = 1/3; RBD = 3·10-7 m2W/K
k_Nan with Kp = 30 W/m·K; <cos2Θ> = 1/3; RBD = 3·10-7 m2W/K
k_Nan with Kp = 600 W/m·K; <cos2Θ> = 0.75; RBD = 3·10-7 m2W/K
k_Nan with Kp = 300 W/m·K; <cos2Θ> = 0.75; RBD = 3·10-7 m2W/K
k_Nan with Kp = 30 W/m·K; <cos2Θ> = 0.75; RBD = 3·10-7 m2W/K
k_Nan with Kp = 600 W/m·K; <cos2Θ> = 1; RBD = 3·10-7 m2W/K
k_Nan with Kp = 300 W/m·K; <cos2Θ> = 1; RBD = 3·10-7 m2W/K
k_Nan with Kp = 30 W/m·K; <cos2Θ> = 1; RBD = 3·10-7 m2W/K

(b).

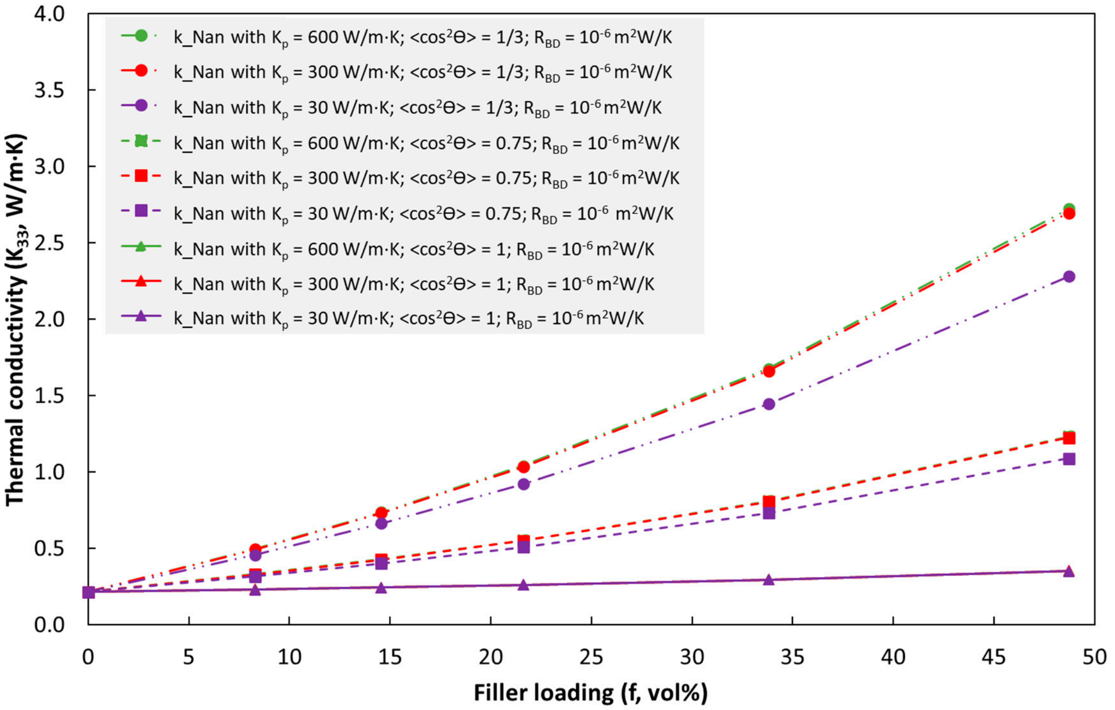

(c).

Figure S12. Effects of K_P, $\langle cos^2\theta \rangle$ and R_{BD} on the thermal conductivity K_{33} of the model of Nan et al. (Eq. S1). Other parameters: K_m = 0.22 W/m·K; p = 0.05; a_3 = 1 µm; Note that, for $\langle cos^2\theta \rangle$ = 1, the curves for K_P = 30; 300 and 600 W/m·K overlap. Also, for $\langle cos^2\theta \rangle$ = 0.75 and 1/3 the curves for K_P = 300 and 600 W/m·K overlap.

To complement the comparison between this model and experimental data for injection moulded composites in the article, Figure S13 compares the model with experimental data for cast composites. In this figure, the lower and upper experimental values of $\langle cos^2\theta \rangle$ (0.4 and 0.65) were used in the model. For the data up to about 34 vol% filler, the agreement between model and experiments is OK, if we assume that the average $\langle cos^2\theta \rangle$ through the disc thickness is somewhat less than 0.4 (since it may be lower in the core than at the surface, at which the XRD measurements was performed) and the effective R_{BD} value is much lower than 10^{-6} (here "effective" means that there is some filler-filler contact in the specimens which is not accounted for in the model). However, at the highest filler loading (about 38 vol%), the model clearly underpredicts the experiment, if parameters assumedly represent an upper limit conductivity for this case ($\langle cos^2\theta \rangle$ = 1/3 and R_{BD} = 0). Hence, at this loading there seems to be a percolation effect in the experimental data, if we believe this model is correct at lower loadings.

S12. The model of Ordóñez-Miranda et al.

Ordóñez-Miranda et al. [11] combined the Nan model above with the Bruggeman integration principle. The resulting model is claimed to be better at high particle volume fractions than the Nan model. However, Ordóñez-Miranda et al. only considered the case with random orientation of particles.

The Ordóñez-Miranda ("O-M") model was implemented by solving Eq. 11 in ref. [11] in combination with Eq. S1b-S1h above (with $\langle cos^2\theta \rangle = 1/3$).

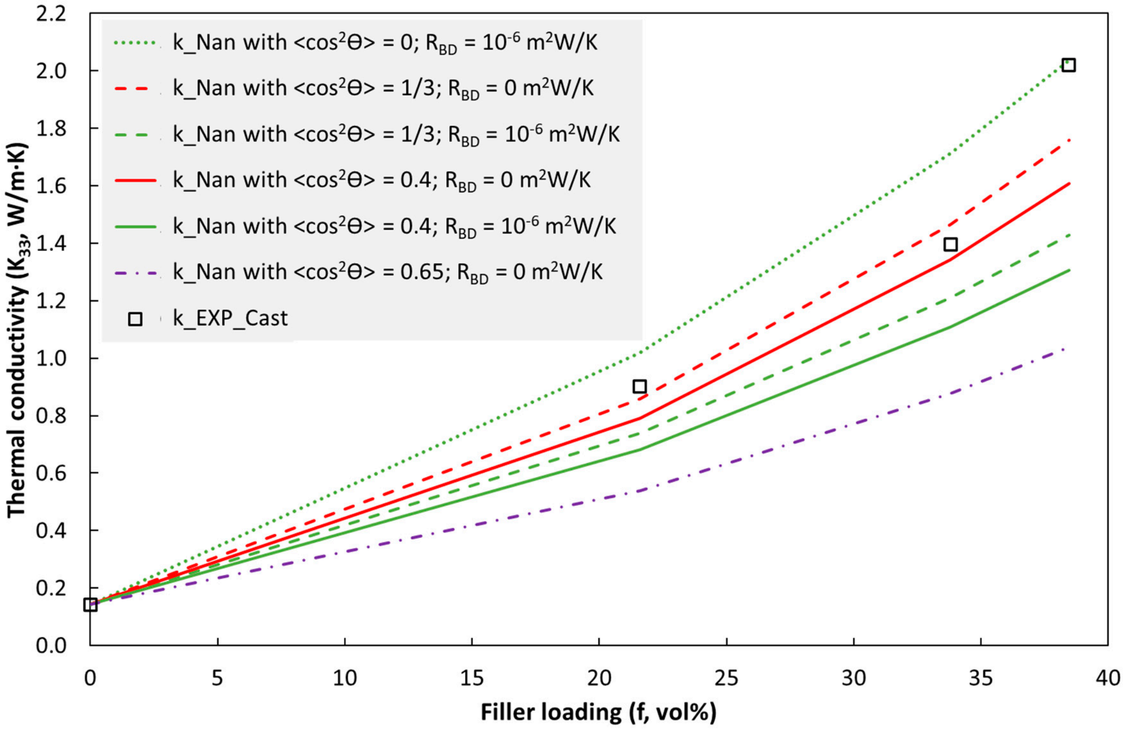

Figure S13. The model of Nan et al. compared to measured thermal conductivities of cast BN3/epoxy composites (k_EXP_Cast). In addition to the model parameters given in the legend, the model parameters were K_m = 0.14 W/m·K, K_p = 300 W/m·K, p = 0.05 and a_3 = 1 µm. (⟨$\cos^2\theta$⟩ = 0.4 and 0.65 are the lower and upper limits of the experimental data obtained by XRD).

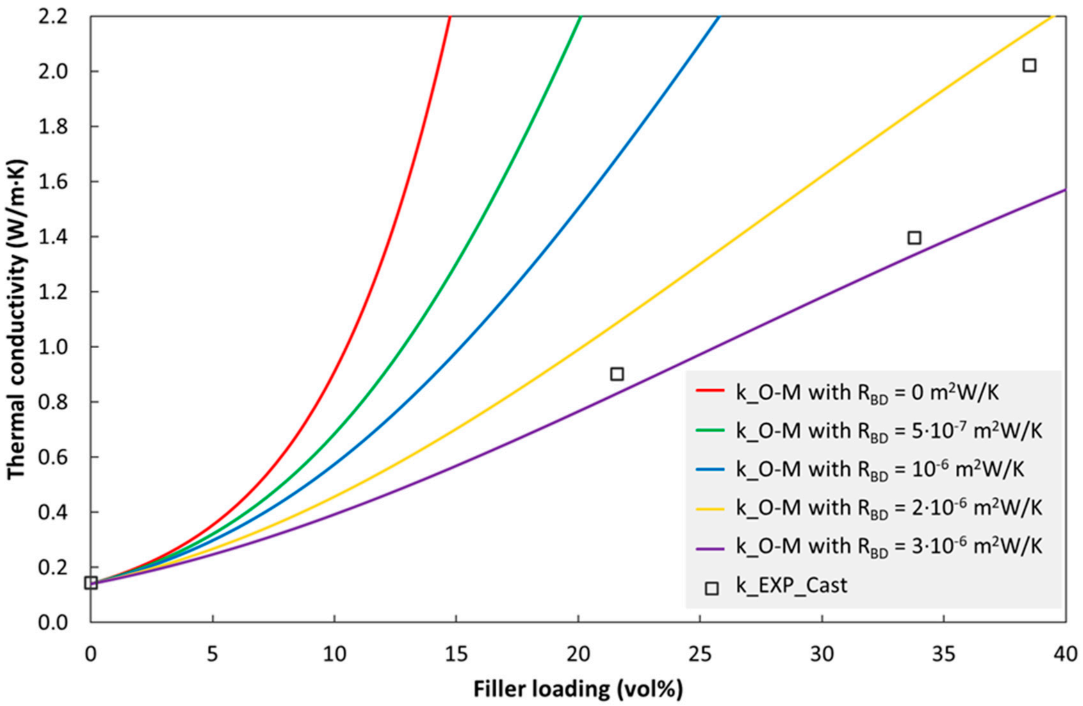

Figure S14. Ordóñez-Miranda model (k_O-M) compared to measured values for cast BN3/epoxy composites (k_EXP_Cast). In addition to the model parameters given in the legend, the model parameters were K_m = 0.14 W/m·K, K_p = 300 W/m·K, p = 0.05, a_1 = 20 µm, a_3 = 1 µm, and ⟨$\cos^2\theta$⟩ = 1/3.

S13. The Model of Sun et al.

Based on a finite element model, Sun et al. [12] derived an analytical model for the thermal conductivity of hBN/polymer composites. In addition to the effects included in the Nan model above, the Sun model also takes into account the anisotropic thermal conductivity of the hBN platelets. Hence, in principle it should be more accurate than the Nan model in section S11 above. However, the Sun model has the same limitations as the models above regarding no filler-filler contact and a homogeneous platelet distribution.

The model of Sun et al. is given by Eqs. S2-S4 below. Note that the expression for K_y' contained an error in ref. [12]. The corrected expression is given below (correction received from Dr. Sun, the first author of ref. [12]).

$$\begin{bmatrix} K_{xx} & K_{xy} & K_{xz} \\ K_{yx} & K_{yy} & K_{yz} \\ K_{zx} & K_{zy} & K_{zz} \end{bmatrix} = C^{-1} \begin{bmatrix} K_x' & 0 & 0 \\ 0 & K_y' & 0 \\ 0 & 0 & K_z' \end{bmatrix} C \quad \text{(S2)}$$

$$\frac{K_x'}{\varepsilon_{p,x}\varepsilon_{t,x}K_m} = 1 + \left[\frac{K_{f,in}f^8 + 3(a+9)}{2(3+700K_{f,in}R_{BD})} - 1\right] f$$

$$\frac{K_y'}{\varepsilon_{p,y}\varepsilon_{t,y}K_m} = 1 + \left[\frac{9K_{f,in}f^8 + \sqrt{3}(a+9)}{2+700K_{f,in}R_{BD}} - 1\right] f \quad \text{(S3)}$$

$$\frac{K_z'}{\varepsilon_{p,z}\varepsilon_{t,z}K_m} = 1 + \frac{3}{2}\left[\frac{5K_{f,o}f^3 + \sqrt{3}(1+1/a)}{1+10^6K_{f,o}R_{BD}} - 1\right] f$$

with

$$\varepsilon_{p,x} \approx 1.00\sim1.05;\ \varepsilon_{p,y} \approx 0.99\sim1.06;\ \varepsilon_{p,z} \approx 1.00$$
$$\varepsilon_{t,x} \approx 0.93\sim1.00;\ \varepsilon_{t,y} \approx 0.99\sim1.05;\ \varepsilon_{t,z} \approx 1.00\sim1.06$$

$$C = \begin{bmatrix} cos\alpha cos\gamma + sin\alpha sin\beta sin\gamma & -sin\alpha cos\beta & -cos\alpha sin\gamma + sin\alpha sin\beta cos\gamma \\ sin\alpha cos\gamma - cos\alpha sin\beta sin\gamma & cos\alpha cos\beta & -sin\alpha sin\gamma + cos\alpha sin\beta cos\gamma \\ cos\beta sin\gamma & sin\beta & cos\beta cos\gamma \end{bmatrix} \quad \text{(S4)}$$

where

K_{xx}, K_{yy}, K_{zz} are thermal conductivities of the hBN/polymer composites along the x, y, z axes.

K_x', K_y', K_z' are the thermal conductivities of the homogenized hBN/polymer composite along three directions in the ideal uniform dispersion model.

K_m is the thermal conductivity (W/m·K) of the polymer matrix

$K_{f,in}$ and $K_{f,o}$ are the thermal conductivity of hBN in the in-plane direction (100 – 1000 W/m·K) and through-plane direction (2 – 40 W/m·K), respectively. According to ref. [12], the K_{zz} value of the Sun model is almost independent of the thermal conductivity of the filler hBN, when the in-plane thermal conductivity of hBN is in the range of (100 – 1000) W/m·K, and the through-plane thermal conductivity of hBN is in the range (2 – 40) W/m·K [12]. Therefore, in our study, the values 300 W/m·K and 2 W/ m·K were used for the in-plane ($K_{f,in}$) and through-plane ($K_{f,o}$) conductivities of h-BN, respectively.

a is the diameter to thickness ratio of the hBN platelets (the inverse of p in the EMA model).

f is volume fraction of filler (vol%)

R_{BD} is the interfacial thermal resistance between filler and polymer matrix.

$\varepsilon_{p,x}$, $\varepsilon_{p,y}$, $\varepsilon_{p,z}$ are position correction factors along the x, y and z axis (all are close to 1)

$\varepsilon_{t,x}$, $\varepsilon_{t,y}$, $\varepsilon_{t,z}$ are tilt angle correction factors (all are close to 1)

In our study, all the correction factors ($\varepsilon_{p,x}$, $\varepsilon_{p,y}$, $\varepsilon_{p,z}$, $\varepsilon_{t,x}$, $\varepsilon_{t,y}$, $\varepsilon_{t,z}$) were set to 1 for simplifying the calculations.

α, β, γ are the tilt angle deviations of the hBN platelets in three directions; γ in the Sun model corresponds to θ in the Nan model.

K_{zz} in the Sun model [12] corresponds to the through-plane conductivity of the Nan model above. Eqs. S2-S4 were calculated with a Python code in our study. The conductivity K_{zz} in the thickness direction of the disc was calculated by averaging over the angles α and β, for a given γ value. Our calculations were validated by comparing with the data in Figures 7 and 10 in ref. [12]. The values were similar, except small difference at volume fractions above 60% in Figure 10b of ref. [12]. The reason for this is unclear.

The Sun model generally predicts lower conductivities than the Nan model. For the cast specimens, the Sun model underestimates the conductivity for all three data points in the interval 20-40 vol% hBN, see Figure S15, but the model may give fair predictions for loadings up to 10 vol%. As for the Nan model, the experimental data has a larger curvature vs. filler loading (vol% hBN) than the model predictions.

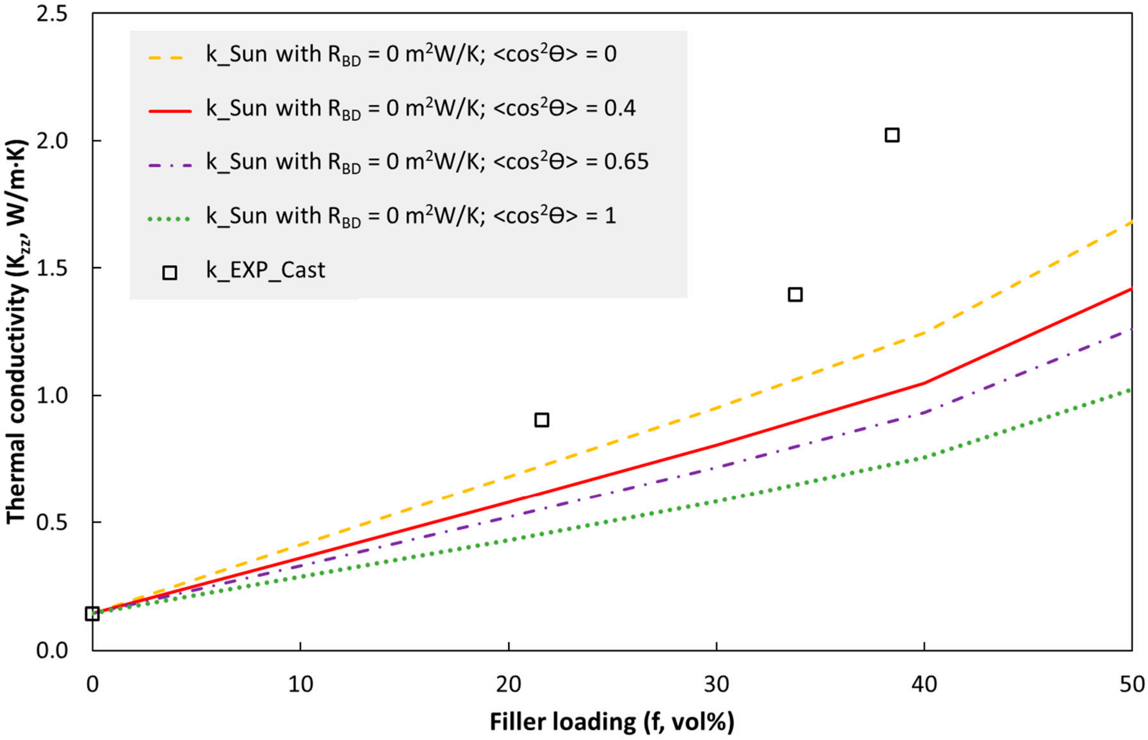

Figure S15. The Sun model (k_Sun) compared with the measured values for cast BN3/epoxy composites (k_EXP_Cast). Parameters used in the model are K_m = 0.14 W/m·K; $K_{f,in}$ = 300 W/m·K and $K_{f,o}$ = 2 W/m·K; a = 20; R_{BD} = 0 m²W/K; and γ equal to θ given by $\langle cos^2\theta \rangle$ = 0, 0.4, 0.65, and 1 (0.4 and 0.65 are the lower and upper limits of the experimental data obtained by XRD).

S14. The Lewis-Nielsen Model

The Lewis-Nielsen model for the thermal conductivity is given by Eq. S5 [13]:

$$k = k_m \left[\frac{1 + fA\xi}{1 - f\xi\psi}\right] \quad \text{(S5a)}$$

$$\xi = \frac{k_p/k_m - 1}{k_p/k_m + A} \quad \text{(S5b)}$$

$$\psi = 1 + f\left[\frac{1 - f_m}{f_m^2}\right] \quad \text{(S5c)}$$

Here f is the particle volume fraction, A is a particle geometry factor, f_m is the maximum volume fraction (often 0.8-0.85 for platelets [13]). The geometry factor represents the effective particle shape in the direction in which the conductivity is measured. Hence, the average particle orientation is embedded in this geometry factor. The drawback of this model is that the geometry factor is mainly an empirical fitting parameter.

In Figure S16, the Lewis-Nielsen model is compared to experimental data for injection moulded specimens. One set of model parameters (A and f_m) can not describe the experimental dataset, if f_m is in the interval stated above. An increase in A is with increasing particle volume fraction is in line with the formation of conducting "chains" of particles as discussed in the article. If a lower value of f_m is used, the model gives a better fit to the experimental data, see Figure S17, but this has no physical basis.

In Figure S18, the Lewis-Nielsen model is compared to experimental data for cast specimens. Note that a best fit yields almost the same f_m as for the injection moulded specimens, but this is again unphysical and due to the overfitting to capture the last data point. The Lewis-Nielsen model can fit the overall non-linearity of the experimental data set with relevant f_m values, but there are only four data points, and the three first data points probably follow a roughly linear trend, while the last point is higher than this trend due to the formation of "chains" of platelets.

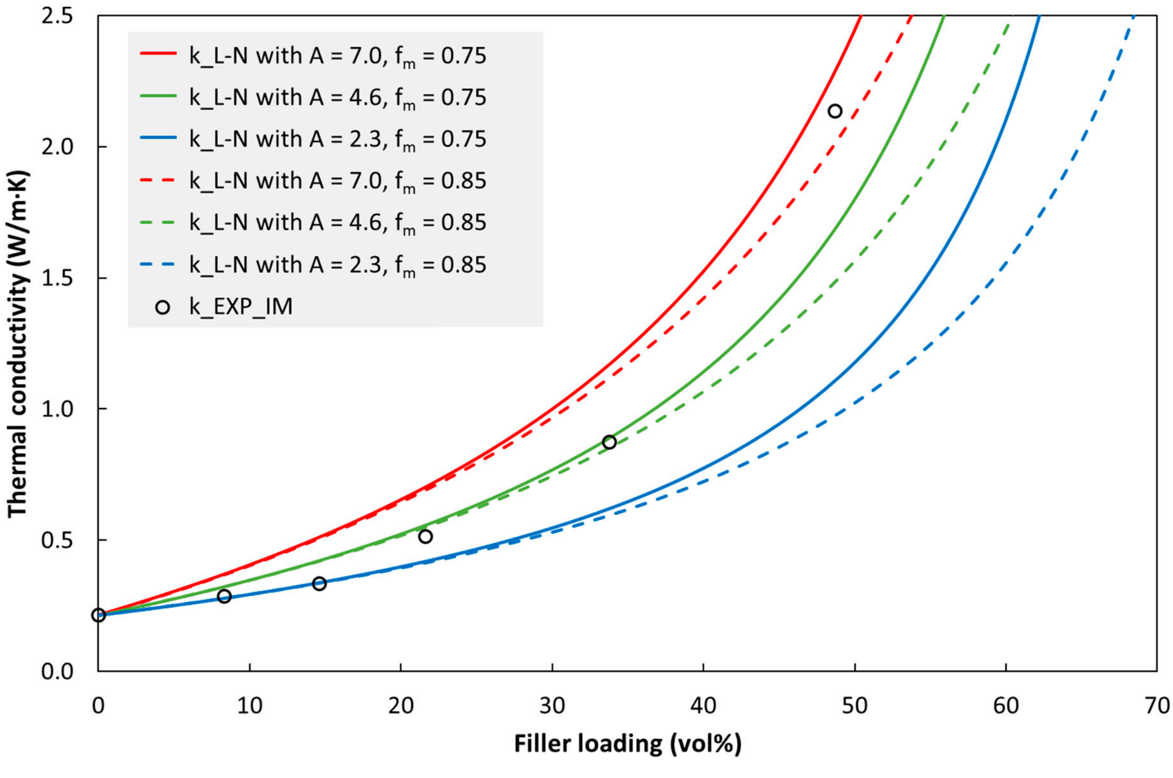

Figure S16. The Lewis-Nielsen model (k_L-N) and experimental data for injection moulded specimens of BN3/TPU (k_EXP_IM). Other parameters: k_m = 0.22 W/m·K and k_p = 300 W/m·K.

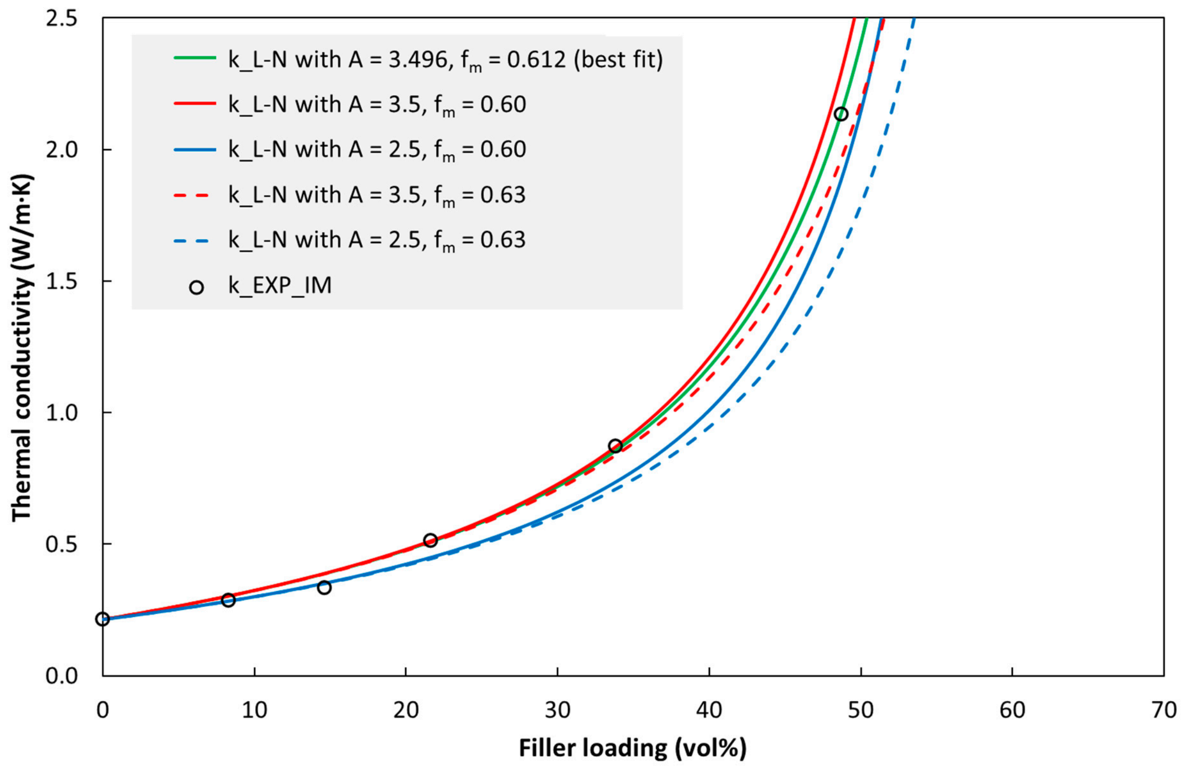

Figure S17. The Lewis-Nielsen model (k_L-N) and experimental data for injection moulded specimens of BN3/TPU (k_EXP_IM). Other parameters: k_m = 0.22 W/m·K and k_p = 300 W/m·K.

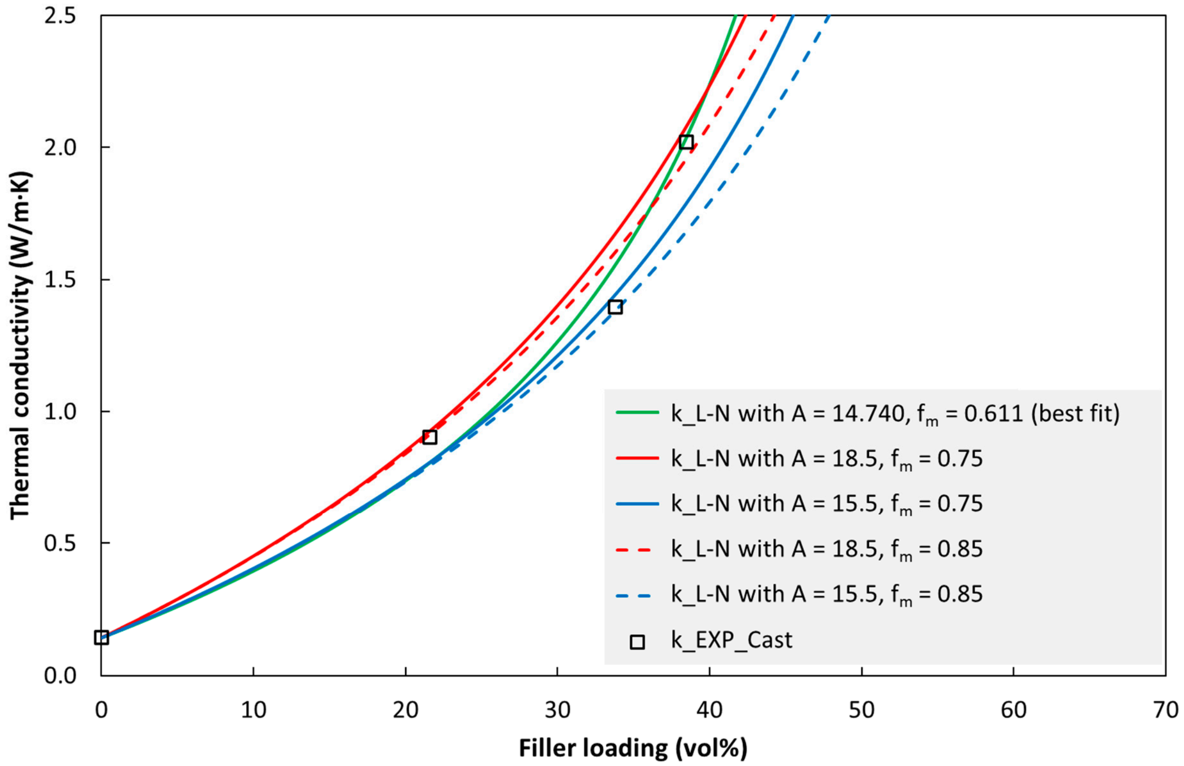

Figure S18. The Lewis-Nielsen model (k_L-N) and experimental data for cast specimens of BN3/epoxy (k_EXP_Cast). Other parameters: k_m = 0.14 W/m·K and k_p = 300 W/m·K.

References

1. Henze Boron Nitride Products AG, "HeBoFill ® LL-SP 120," *Technical Datasheet*, p. 8374, 2020.
2. Henze Boron Nitride Products AG, "HeBoFill ® CL-ADH 020," *Technical Datasheet*, pp. 49–50, 2020.
3. H.-C. Tseng, R.-Y. Chang, and C.-H. Hsu, "Comparison of recent fiber orientation models in injection molding simulation of fiber-reinforced composites," *Journal of Thermoplastic Composite Materials*, vol. 33, no. 1, pp. 35–52, Jan. 2020, doi: 10.1177/0892705718804599.
4. S.M. Mazahir, G.M. Velez-Garcia, P. Wapperom, and D. Baird, "Fiber orientation in the frontal region of a center-gated disk: Experiments and simulation," *J Nonnewton Fluid Mech*, vol. 216, pp. 31–44, Feb. 2015.
5. C. W. Nan, R. Birringer, D. R. Clarke, and H. Gleiter, "Effective thermal conductivity of particulate composites with interfacial thermal resistance," *J Appl Phys*, vol. 81, no. 10, pp. 6692–6699, 1997, doi: 10.1063/1.365209.
6. C. Yuan, B. Duan, L. Li, B. Xie, M. Huang, and X. Luo, "Thermal Conductivity of Polymer-Based Composites with Magnetic Aligned Hexagonal Boron Nitride Platelets," *ACS Appl Mater Interfaces*, vol. 7, no. 23, pp. 13000–13006, 2015, doi: 10.1021/acsami.5b03007.
7. Y. Xue, X. Li, H. Wang, F. Zhao, D. Zhang, and Y. Chen, "Improvement in thermal conductivity of through-plane aligned boron nitride/silicone rubber composites," *Mater Des*, vol. 165, p. 107580, 2019, doi: 10.1016/j.matdes.2018.107580.
8. C. Pan, J. Zhang, K. Kou, Y. Zhang, and G. Wu, "Investigation of the through-plane thermal conductivity of polymer composites with in-plane oriented hexagonal boron nitride," *Int J Heat Mass Transf*, vol. 120, no. December, pp. 1–8, 2018, doi: 10.1016/j.ijheatmasstransfer.2017.12.015.
9. H. Chen, V. v. Ginzburg, J. Yang, Y. Yang, W. Liu, Y. Huang, L. Du, and B. Chen, "Thermal conductivity of polymer-based composites: Fundamentals and applications," *Prog Polym Sci*, vol. 59, pp. 41–85, 2016, doi: 10.1016/j.progpolymsci.2016.03.001.
10. J. Liu, W. Li, Y. Guo, H. Zhang, and Z. Zhang, "Improved thermal conductivity of thermoplastic polyurethane via aligned boron nitride platelets assisted by 3D printing," *Compos Part A Appl Sci Manuf*, vol. 120, no. February, pp. 140–146, 2019, doi: 10.1016/j.compositesa.2019.02.026.
11. J. Ordóñez-Miranda, J. J. Alvarado-Gil, and R. Medina-Ezquivel, "Generalized Bruggeman Formula for the Effective Thermal Conductivity of Particulate Composites with an Interface Layer," *Int J Thermophys*, vol. 31, no. 4–5, pp. 975–986, May 2010, doi: 10.1007/s10765-010-0756-2.
12. Y. Sun, L. Zhou, Y. Han, L. Cui, and L. Chen, "A new anisotropic thermal conductivity equation for h-BN/polymer composites using finite element analysis," *Int J Heat Mass Transf*, vol. 160, p. 120157, 2020, doi: 10.1016/j.ijheatmasstransfer.2020.120157.
13. C. Heinle, Z. Brocka, G. Hülder, G. Ehrenstein, and T. Osswald, "Thermal conductivity of polymers filled with non-isometric fillers: A process dependent, anisotropic property," in *Proceedings of the 67th Annual Technical Conference of the Society of Plastics Engineers (ANTEC 2009, Chicago)*, Jun. 2009, pp. 883–889.
